# Comparisons of 25 cerebrospinal fluid cytokines in a case–control study of 106 patients with recent-onset depression and 106 individually matched healthy subjects

**DOI:** 10.1186/s12974-023-02757-2

**Published:** 2023-04-04

**Authors:** Nina Vindegaard Sørensen, Nis Borbye-Lorenzen, Rune Haubo Bojesen Christensen, Sonja Orlovska-Waast, Rose Jeppesen, Kristin Skogstrand, Michael Eriksen Benros

**Affiliations:** 1 Copenhagen Research Centre for Mental Health, Mental Health Centre Copenhagen, Copenhagen University Hospital, Gentofte Hospitalsvej 15, 4. Sal, 2900 Hellerup, Denmark; 2 Department of Immunology and Microbiology, Faculty of Health and Medical Sciences, University of Copenhagen, Copenhagen, Denmark; 3 Center for Neonatal Screening, Department of Congenital Disorders, Statens Serum Institut, Copenhagen, Denmark

**Keywords:** Depression, Immunology, Cerebrospinal fluid, Biomarkers, Cytokines, Chemokines

## Abstract

**Background:**

Neuroinflammation has been suggested as a contributor to the pathophysiology of depression; however, large case–control studies investigating cytokine levels in the cerebrospinal fluid (CSF) from patients with recent-onset depression by multiplex analyses are missing.

**Methods:**

An individually matched (sex and age) prospective case–control study comparing patients with recent-onset depression to healthy controls. CSF was analyzed with the Mesoscale V-PLEX Neuroinflammation Panel 1. *Outcomes*: comparisons of analyte levels in the CSF between groups with interleukin (IL)-6 and IL-8 as primary outcomes and 23 other cytokines as secondary outcomes.

**Results:**

We included 106 patients (84.0% outpatients) with recent-onset depression and 106 healthy controls. There were no significant differences in the primary outcomes IL-6 (relative mean difference (MD): 1.10; 95% confidence interval (CI) 0.93–1.30; *p* = 0.276) or IL-8 levels (MD: 1.05; 95% CI 0.96–1.16; *p* = 0.249) relative to healthy controls. IL-4 was 40% higher (MD: 1.40; 95% CI 1.14–1.72; *p* = 0.001), monocyte chemoattractant protein (MCP)-1 was 25% higher (MD: 1.25; 95% CI 1.06–1.47; *p* = 0.009) and macrophage inflammatory protein (MIP)-1β was 16% higher (MD: 1.16; 95% CI 1.02–1.33; *p* = 0.025) in patients with depression relative to healthy controls. However, only IL-4 was significantly elevated after correction for multiple testing of secondary outcomes (*p* = 0.025).

**Conclusion:**

We found no significant differences in CSF levels of the co-primary outcomes IL-6 and IL-8, however, the higher CSF levels of IL-4, MCP-1 and MIP-1β among patients with recent-onset depression compared to healthy controls indicate a potential role of these cytokines in the neuroinflammatory response to depression.

**Supplementary Information:**

The online version contains supplementary material available at 10.1186/s12974-023-02757-2.

## Background

Inflammation is a proposed contributor to the pathophysiology of depression, however, the potential contribution of neuroinflammatory mechanisms to depression is still not fully elucidated [1]. Biomarkers of neuroinflammation include cytokines (here used as a term referring to cytokines, interferons (INF), interleukins (IL), chemokines, and the tumor necrosis factor (TNF) family [2]) that are key modulators of inflammation secreted from a broad variety of cells (both immune cells and others) [3]. A meta-analysis of cytokine alterations in the blood, based on 107 studies comprising a total of 5,166 patients with depression compared to 5,083 healthy controls, found levels of IL-3, IL-6, IL-12, IL-18 and TNF-ɑ to be higher in blood from the patients with depression [4]. A meta-analysis investigating the impact of the phase of depression found IL-6 levels in blood to be increased only in the acute phase of illness and among patients with chronic depression (based on 18 and 12 studies, respectively) [5]. However, due to the blood–brain barrier (BBB) [6] biomarkers measured in the cerebrospinal fluid (CSF) more directly reflect neuroinflammation, and in contrary to peripheral cytokine levels measured in the blood, CSF cytokine levels in recent-onset depression are not extensively investigated.

In a recently published study from our group investigating routine neuroinflammatory markers measured in CSF, we found patients with recent-onset depression to have 18% higher white cell count in the CSF compared to the healthy controls, indicating a neuroinflammatory response to depression [7]. A meta-analysis of immune markers in the CSF found levels of the cytokines IL-6, IL-8 and TNF-ɑ to be higher in patients with depression as compared to healthy controls [8]. However, whereas a more recent meta-analysis confirmed the finding of higher levels of CSF IL-6, no group differences regarding IL-8 and TNF-ɑ were seen when comparing patients with depression to healthy controls [9]. Since the first meta-analysis was conducted [8], the hitherto largest study evaluating cytokines by multiplex in CSF has been published comprising 104 patients with depression and 118 healthy controls [10]. This study reported no significant group differences of CSF IL-8 levels and did not evaluate CSF IL-6 or TNF-ɑ [10]. Noteworthy, the mean age of patients was 43.4 years, their duration of illness ranged from 0 to 38 years and the mean HDRS-21 score was 11.3 (9.2) [10]. Previous studies of IL-6 in CSF from patients with depression compared to healthy controls included 32 patients as a maximum [9]. Thus, large-scale studies of patients with recent-onset depression of moderate severity compared to individually matched healthy controls are still missing in the context of CSF cytokines related to depression.

The aim of the present study was to evaluate differences in cytokine levels in the CSF from a large cohort of patients with recent-onset depression compared to healthy controls individually matched on sex and age, hereby investigating the hypothesis that patients with depression have higher levels of CSF pro-inflammatory cytokines relative to healthy controls.

## Materials and methods

This study was designed as a prospective case–control study including 106 patients with first time depression (International Classification of Diseases 10th Revision (ICD-10): F32.0) and 106 individually matched (age and sex) healthy controls. A detailed protocol paper has been published elsewhere prior to data-analyses [11]. Data on routine measurements have previously been published elsewhere [7]. All participants were included for research purpose only and all lumbar punctures were conducted for research purpose, also for the healthy control group.

### Outcomes

The two co-primary outcomes of this study were IL-6 and IL-8 levels in CSF from patients with recent-onset depression compared to healthy controls. The secondary outcomes were levels of IFN-γ, IL-1α, IL-1β, IL-2, IL-4, IL-5, IL-7, IL-10, IL-12/IL-23p40, IL-13, IL-15, IL-16, IL-17A, interferon gamma-induced protein-10 (IP-10), monocyte chemoattractant protein (MCP)-1, MCP-4, macrophage-derived chemokine (MDC), macrophage inflammatory protein (MIP)-1α, MIP-1β, thymus- and activation-regulated chemokine (TARC), TNF-α, TNF-β and intercellular adhesion molecule-1 (ICAM-1) measured in CSF from patients with recent-onset depression compared to healthy controls.

### Participants

From October 2018 until April 2021 patients were recruited from in- and out-patient facilities of the Mental Health Services of the Capital Region and Region Zealand of Denmark. Patients had a first-time diagnosis of depression (according to ICD-10: F32) diagnosed within the past year with ongoing depressive symptoms and were aged between 18 and 50 years. Patients were excluded if they had a previous psychiatric diagnosis within ICD-10 from F20 to F39, contraindications to lumbar puncture, conditions or medication with known substantial impact on the immune system, except psychotropics as needed for the current depression and as specified in the Study Protocol [11] or conditions other than depressive symptoms with impact on WHO Schedules for Clinical Assessment in Neuropsychiatry (SCAN) interview (Version 2.1) [12]. Individually age and sex matched, mentally and physically healthy controls from the same geographical area were recruited mainly by internet advertisement in the period from September 2018 to July 2021. Exclusion criteria for healthy controls were as for patients, and additionally, healthy controls were excluded prior to lumbar puncture if SCAN interview revealed present or previous psychiatric illness. An overview of minor somatic co-morbidities is found in Additional file 1: Table S1.

### Clinical assessment

Patients were diagnosed at the mental health center responsible for treatment and the diagnoses were confirmed by SCAN interview conducted as part of the enrollment. The 17-item Hamilton Depression Rating Scale (HDRS-17) [13] and the 10-item Montgomery–Asberg Depression Rating Scale (MADRS-10) [14] were used to assess severity of depressive symptoms. Hamilton Anxiety Rating Scale (HARS) [15] was used to rate anxiety symptoms and Positive and Negative Symptom Scale (PANSS) [16] to rate psychotic symptoms. Global Assessment of Functioning (GAF) [17] assessed level of functioning and cognitive testing was performed by using the Brief Assessment of Cognition in Schizophrenia (BACS) [18], Montreal Cognitive Assessment (MoCA) [19] and Mini-Mental State Examination (MMSE) [20]. Prior to lumbar puncture, all participants had a neurological examination including Neurological Evaluation Scale for evaluation of Neurological Soft Signs (NSS) [21].

### Cytokine multiplex analyses

CSF samples were collected by the use of an atraumatic needle 22G with the participant placed in decubitus position in most cases. The CSF collection was carried out according to the study protocol [11] based on current consensus [22]. CSF samples were collected between 9:55 a.m. and 12:00 p.m. (90%) and stored at -80̊C until analyses and mean time from sample collection (first droplet) until freezing was 75.9 (15.9) minutes. CSF samples were analyzed at Section for Biomarkers, Immunology and Antibodies, Center for Neonatal Screening, Statens Serum Institut, Artillerivej 5, DK-2300 Copenhagen, Denmark, by the V-PLEX Neuroinflammation Panel 1 (#K15210D, MesoScale (MSD), Rockville, USA) on the Meso QuickPlex SQ 120 (MSD). To minimize intrasample variation, the first aliquot collected after centrifugation was used for all the analyses. CSF (170 μL) was aliquoted to two set of six 96-well plates (using 340 μL CSF/sample) and plates were stored at -80̊ C until analysis. Coefficients of intra- and inter-assay variations are found in Additional file 1: Table S2. All plates were analyzed in accordance with manufacturer’s instruction; for further details see Additional file 1: eMethods. All laboratory personnel were blinded to case–control status. As prespecified in the protocol published prior to data-analyses we only included 25 of the 37 cytokines measured by V-PLEX Neuroinflammation Panel 1 in the present study [11]. These 25 cytokines were chosen as they are all thought to be potentially involved in a low-grade inflammatory response [3]. Thus, this study does not evaluate markers related to angiogenesis and vascular injury (including vascular endothelial growth factor), however, ICAM-1 was included due to the involvement in various immunological processes [23].

### Statistics

Power and sample size calculations based on two-sample *t*-tests led to the expectation of good power (> 80%) for IL-6 and low power for IL-8 (< 50%) based on previous studies [11]. Baseline characteristics were assessed by either Welch two-sample *t*-tests (continuous data) or Pearson *χ*^2^ tests (categorical data). Categorical variables are shown in absolute numbers and percentages. Continuous variables are shown as mean (SD). Based on previous studies and information from the MesoScale company, we expected a proportion of biomarker measurements to be lower than the lower limit of detection (LLOD) and a larger proportion to be lower than the lower limit of quantification (LLOQ). Thus, censored log-normal models [24, 25] adjusted for sex, age and plate were applied for primary and secondary analyses treating measurements below the lower limit of detection (LLOD) as censored and as a sensitivity analysis treating measurements below the LLOQ as censored (see eMethods for details). Plate number was adjusted for, since a considerable variation in percentage of detected and quantifiable samples was observed between plates for some of the outcome measures, and as cases and controls were not randomly distributed between plates (Additional file 1: Figs. S1 and S2). LLOD and LLOQ are provided in Additional file 1: Table S3. Effect estimates are given as relative mean difference (MD) between groups with 95% confidence intervals (95%CIs). Sensitivity analyses accounting for time from sample collection to freezing as well as storage duration were also conducted. Analyses of cytokines categorized by properties included the following categories: pro-inflammatory, anti-inflammatory, innate, adaptive, acute, chronic (humoral/cellular), and T helper cell response (Th1, Th2, Th17, Treg) and were all based on previous peer-reviewed papers (Additional file 1: Table S4). For categorized analyses, cytokine measurements were standardized on the log-scale using mean and SD from censored log-normal models. In a second step, the standardized cytokines in each category were combined to form a single category-specific variable, which, in a third step, was used to compare subjects with depression to healthy controls while adjusting for sex, age and plate. To account for samples being differentially censored below LLOD across cytokines, the second and third steps were performed jointly in a censored log-normal model using weighting giving equal weight to each cytokine in a category. Previous studies indicated altered levels of IL-6 and IL-8 to be related to severity of depressive symptoms [26, 27], anxiety symptoms [28] and suicide attempts [27]. Therefore, subgroup analyses of cytokine levels above LLOD and based on severity of depression (assessed by HDRS-17), severity of co-morbid anxiety (assessed by HARS) and suicidal ideation (assessed by SCAN interview) were performed post hoc similar to the primary models but including the interaction between group and the subgroup variable. These analyses were also conducted for significant secondary outcomes. Furthermore, subgroup analyses on smoking status, use of antidepressants and antipsychotics were also performed for primary and significant secondary outcomes. Two-sided *p*-values < 0.05 were considered significant and adjustment for multiple testing for secondary outcomes was done using Holm’s sequential Bonferroni Method methods. All analyses were performed in the program R (version 4.0.5) [29].

**Fig. 1:**
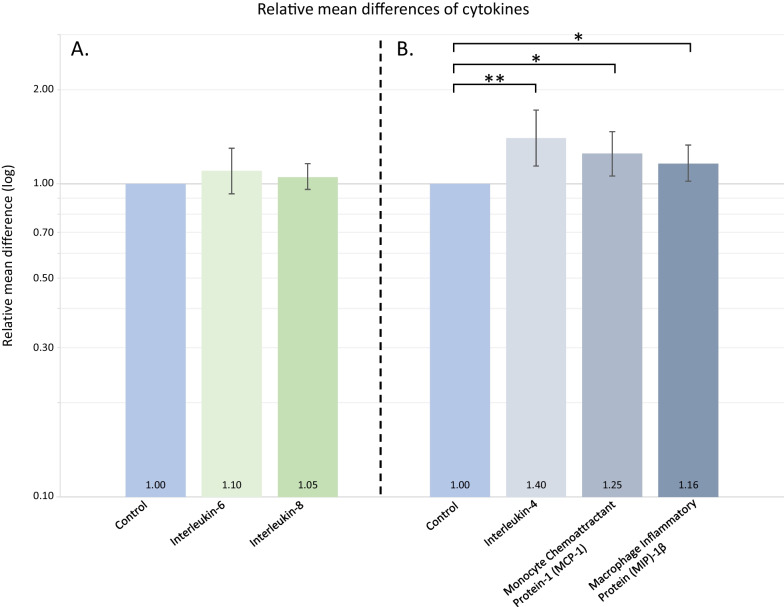
The figure illustrates the results of the primary outcomes and the secondary outcomes that were significantly altered. Relative mean differences are based on measurements above lower limit of detection (LLOD) from Statens Serum Institute. **A** There were no significant differences in the relative mean difference of IL-6 and IL-8 levels between patients with depression as compared to healthy controls. **B** IL-4 levels were higher among patients with depression also after correction of multiple testing, whereas findings of MCP-1 and MIP-1β did not survive correction for multiple testing. *p < 0.05 prior to correction for multiple testing. **p < 0.05 after correction for multiple testing. *IL* interleukin. *LLOD* lower limit of detection. *MCP* monocyte chemoattractant protein. *MIP* macrophage inflammatory protein

**Fig. 2:**
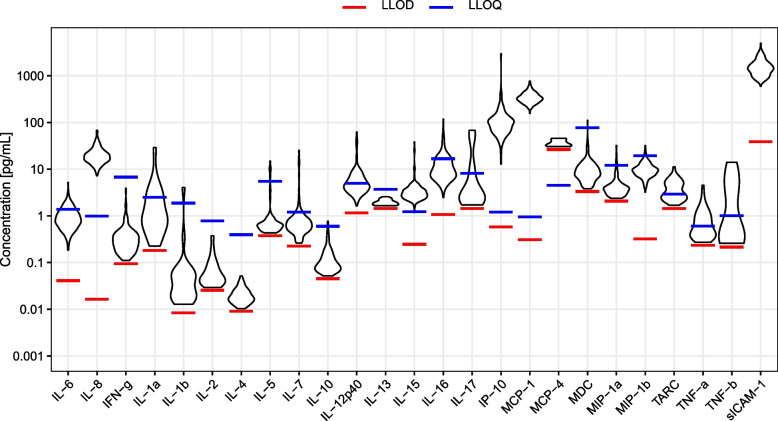
The figure illustrates the distribution of concentrations of all outcome measurements related to the lower limit of detection (LLOD) from Statens Serum Institute and lower limit of quantification (LLOQ) from MesoScale. As seen, the proportion of samples below LLOD is substantial to some outcomes (e.g., MCP-4 and TNF-β), whereas other outcomes are above LLOD in all samples (e.g., IL-6 and IL-8). LLOQ sICAM: 15.0 pg/mL. ICAM: intercellular adhesion molecule. *IFN* interferon. *IL* interleukin. *IP* interferon gamma-induced protein-10. *LLOD* lower limit of detection. *LLOQ* lower limit of quantification. *MCP* monocyte chemoattractant protein. *MDC* macrophage-derived chemokine. *MIP* macrophage inflammatory protein. *TARC* thymus- and activation-regulated chemokine. *TNF* tumor necrosis factor

## Results

In this study, 106 patients with recent-onset depression and 106 age- and sex-matched healthy controls were included. In each group, 71 women and 35 men were included. Mean age of patients was 26.0 (7.6) years and did not differ significantly from healthy controls with a mean age of 26.4 (6.8) years (*p* = 0.701). Among patients, 33.0% were treated with antidepressants and 84.0% were outpatients. Patients had mean HDRS-17, MADRS-10 and HARS scores of 20.6 (6.3), 29.5 (7.6) and 20.7 (9.3), respectively, all significantly higher compared to healthy individuals (all *p* < 0.001). A full overview of demographic characteristics of the population is provided in Table 1.

**Table 1:** Characteristics of the population

	Depression (*N* = 106)	Healthy (*N* = 106)	*p-*value
Females (%)	71 (67.0)	71 (67.0)	1.000
Males (%)	35 (33.0)	35 (33.0)	1.000
Age in years (SD)	26.0 (7.6)	26.4 (6.8)	0.701
BMI (SD)	24.0 (5.5)	23.7 (4.2)	0.698
Weekly use of alcohol in units (SD)	3.2 (3.9)	5.0 (6.5)	0.016
Smokers (%)	32 (30.2)	13 (12.3)	0.001
Antidepressant medication (%)	35 (33.0)	0 (0.0)	< 0.001
Antipsychotic medication (%)	12 (11.3)	0 (0.0)	< 0.001
Time from diagnosis to inclusion, mean weeks (SD)	4.0 (5.5)	NA	NA
Outpatients (%)	89 (84.0)	NA	NA
Rating scale scores^c^			
HDRS-17 (SD)	20.6 (6.3)	1.1 (1.5)	< 0.001
MADRS-10 (SD)	29.5 (7.6)	1.2 (1.7)	< 0.001
HARS (SD)	20.7 (9.3)	2.0 (2.2)	< 0.001
PANSS total (SD)	48.8 (7.2)	31.5 (2.1)	< 0.001
GAF-F (SD)	49.4 (12.9)	88.9 (5.1)	< 0.001
BACS composite^a^ (SD)	− 0.4 (0.6)	0.0 (0.6)	< 0.001
MMSE total (SD)	29.1 (1.2)	29.4 (1.0)	0.053
MoCA (SD)	27.6 (2.0)	28.0 (2.0)	0.143
Serum hs-CRP mg/L (SD)^b^	0.71 (3.66)	0.71 (3.74)	0.980
Serum white blood cell count × 10^9^L (SD)^b^	5.79 (1.29)	5.79 (1.27)	0.977

Categorical variables are shown in absolute numbers and (%). Continuous variables are shown as mean (± SD). *p*-values are based on Pearson’s Chi-square test for categorical variables and Welch two-sample* t*-test for continuous variables

*BACS* Brief Assessment of Cognition in Schizophrenia, *BMI* body mass index, *GAF* Global Assessment of Functioning, *HARS* Hamilton Anxiety Rating Scale, *HDRS*-17 17-item Hamilton Depression Rating scale, *MADRS*-*10* 10-item Montgomery–Asberg Depression Rating Scale, *MMSE* Mini-Mental State Examination, *MoCA* Montreal Cognitive Assessment, *PANSS* Positive and Negative Symptom Scale, *Hs-CRP* high sensitivity C-reactive protein

^a^Reference for z-score: healthy controls

^b^Geometric mean and standard deviation

^c^*p*-value was unchanged by use of Kruskal–Wallis rank sum test instead of Welch two-sample *t*-test

### Primary outcomes: IL-6 and IL-8

There were no significant differences in levels of CSF IL-6 (MD: 1.10; 95% CI 0.93–1.30; *p* = 0.276) or IL-8 (MD: 1.05; 95% CI 0.96–1.16; *p* = 0.294) among patients with depression relative to healthy controls (Fig. 1, Table 2).

**Table 2:** CSF cytokines above LLOD from 106 patients with depression and 106 healthy controls

	Unit	Depression (*N* = 106)		Healthy controls (*N* = 106)		Relative mean difference (95% CI)	*p*-values
		*N* > LLOD	Mean (95% CI)	*N* > LLOD	Mean (95% CI)		
IL-6	10^–12^ g/mL	106	0.90 (0.81–0.99)	106	0.81 (0.73–0.89)	1.10 (0.93–1.30)	0.276
IL-8	10^–12^ g/mL	106	17.69 (16.71–18.72)	106	20.40 (19.27–21.59)	1.05 (0.96–1.16)	0.294
IFN-γ^a^	10^–12^ g/mL	92	0.26 (0.22–0.29)	67	0.16 (0.13–0.18)	1.04 (0.82–1.31)	0.762
IL-1α^a,b^	10^–14^ g/mL	13	0.36 (0.05–2.66)	9	0.21 (0.02–1.79)	1.13 (0.18–7.26)	0.899
IL-1β^a^	10^–14^ g/mL	18	0.05 (0.01–0.22)	8	0.02 (0.00–0.10)	3.52 (0.92 -13.45)	0.066
IL-2^a,b^	10^–14^ g/mL	28	1.11 (0.70–1.76)	15	0.75 (0.43–1.29)	1.49 (0.92–2.41)	0.105
IL-4^a,b^	10^–14^ g/mL	69	1.28 (1.12–1.48)	40	0.92 (0.78–1.08)	1.40 (1.14–1.72)	0.001^c^
IL-5^a^	10^–12^ g/mL	71	0.50 (0.42–0.59)	74	0.55 (0.46–0.65)	0.84 (0.64–1.12)	0.236
IL-7^a^	10^–12^ g/mL	93	0.56 (0.49–0.63)	99	0.66 (0.59–0.75)	0.90 (0.73–1.12)	0.345
IL-10^a^	10^–14^ g/mL	87	8.44 (7.41–9.61)	71	7.11 (6.19–8.16)	1.04 (0.84–1.31)	0.700
IL-12^a,d^	10^–12^ g/mL	104	5.19 (4.69–5.74)	105	4.76 (4.30–5.27)	0.98 (0.82–1.16)	0.792
IL-13^a^	10^–12^ g/mL	12	1.11 (0.94–1.32)	18	1.20 (1.04–1.39)	0.98 (0.84–1.15)	0.803
IL-15	10^–12^ g/mL	106	3.13 (2.90–3.37)	106	3.24 (3.01–3.49)	0.95 (0.84–1.08)	0.454
IL-16	10^–12^ g/mL	106	9.60 (8.75–10.54)	106	11.02 (10.04–12.10)	0.89 (0.76–1.05)	0.164
IL-17A^a,b^	10^–13^ g/mL	15	0.95 (0.27–3.34)	10	0.83 (0.21–3.34)	1.15 (0.40–3.29)	0.795
IP-10^a^	10^–11^ g/mL	106	9.08 (8.04–10.24)	105	9.15 (8.11–10.33)	1.15 (0.93–1.41)	0.191
MCP-1^a^	10^–10^ g/mL	106	3.38 (3.07–3.73)	105	3.12 (2.83–3.44)	1.25 (1.06–1.47)	0.009^e^
MCP-4^a,b^	10^–11^ g/mL	5	1.35 (0.74–2.46)	2	1.13 (0.54–2.35)	1.20 (0.85–1.70)	0.310
MDC^a^	10^–12^ g/mL	94	7.35 (6.61–8.17)	92	7.81 (7.02–8.68)	0.94 (0.78–1.13)	0.497
MIP-1α^a^	10^–12^ g/mL	68	2.72 (2.46–3.02)	82	3.53 (3.20–3.90)	0.87 (0.73–1.03)	0.103
MIP-1β^a^	10^–12^ g/mL	106	9.54 (8.83–10.31)	105	9.00 (8.33–9.72)	1.16 (1.02–1.33)	0.025^e^
TARC^a^	10^–12^ g/mL	59	1.94 (1.69–2.22)	65	2.18 (1.91–2.49)	1.07 (0.86–1.33)	0.559
TNF-α^a^	10^–13^ g/mL	26	1.06 (0.70–1.59)	34	1.36 (0.92–2.02)	0.74 (0.44–1.25)	0.266
TNF-β^a^	10^–14^ g/mL	7	0.15 (0.01–2.00)	8	0.29 (0.02–3.34)	0.31 (0.04–2.29)	0.251
ICAM-1	10^–9^ g/mL	106	1.48 (1.40–1.57)	106	1.41 (1.33–1.50)	1.04 (0.94–1.16)	0.401

Estimated means, relative mean differences, CIs and *p*-values are based on censored log-normal models adjusted for sex, age and plate

*CI* confidence interval, *CSF* cerebrospinal fluid, *LLOD* lower limit of detection, *IFN* interferon, *IP-10* interferon gamma-induced protein-10, *IL* interleukin, *MCP* monocyte chemoattractant protein, *MDC* macrophage-derived chemokine, *MIP* macrophage inflammatory protein, *TARC* thymus- and activation-regulated chemokine, *TNF* tumor necrosis factor, *sICAM* soluble intercellular adhesion molecule

^a^Analyses included censored measurements

^b^Not adjusted for plate due to too few measurements above LLOD

^c^*p*-value after correction for multiple testing: 0.025

^d^IL12/23p40

^e^Not significant after correction for multiple testing

Likewise, no significant differences for IL-6 and IL-8 were observed between groups when censoring measurements below the LLOQ instead of LLOD (Additional file 1: Table S5). All measurements of IL-8 were above LLOQ across groups, whereas this only applied to 11.8% of IL-6 measurements.

All measurements of IL-8 and IL-6 were above LLOD (Fig. 2, Additional file 1: Table S6) and neither intra-assay nor inter-assay variation raised concerns (Additional file 1: Table S2). Summary statistics of observed data are provided in Additional file 1: Table S7.

### Secondary outcomes

IL-4 was 40% higher (MD: 1.40; 95% CI 1.14–1.72; *p* = 0.001) among patients with depression as compared to healthy controls, and still significantly higher after correction for multiple testing (*p* = 0.025). MCP-1 was 25% higher (MD: 1.25; 95% CI 1.06–1.47; *p* = 0.009) and MIP-1β was 16% higher (MD: 1.16; 95% CI 1.02–1.33; *p* = 0.025) among patients with depression as compared to healthy controls, but neither survived correction for multiple testing. No significant differences were found for the remaining 20 secondary outcomes (Table 2). With measurements below LLOQ censored, IL-16 was found to be 53% lower among patients relative to controls (MD: 0.47; 95% CI 0.29–0.78; *p* = 0.003), whereas MCP-1 was 22% higher (MD: 1.22; 95% CI 1.05–1.40; *p* = 0.008) (Additional file 1: Table S5). Only eight (IL-15, IL-16, ICAM-1, IP-10, MCP-1, MIP-1β, IL-12 and IL-7) of the 23 secondary outcome cytokines were above LLOD in more than 90% of samples and only four (IL-15, ICAM-1, IP-10 and MCP-1) were above LLOQ in more than 90% samples across groups (Additional file 1: Table S6), however, neither intra-assay nor inter-assay variation raised concerns (Additional file 1: Table S2).

### Analyses of cytokines categorized by properties

Although the properties of the different cytokines are complex and influenced by temporal aspects of immune activation and potential effects occurring from sampling to centrifugation, we nevertheless attempted to perform categorization of the cytokines by properties into the following categories: pro-inflammatory, anti-inflammatory, innate, adaptive, acute, chronic (humoral and cellular) and T-helper cell response (Additional file 1: Table S4). However, we did not find any significant group differences in the analyses of cytokines categorized by expected properties (Additional file 1: Table S8).

### Sensitivity analyses

For IL-8 and IL-6 levels above LLOD we found no significant differences related to depression severity (assessed by HDRS-17 score > 24 vs ≤ 24), severity of co-morbid anxiety (assessed by HARS score > 20 vs ≤ 20) or related to suicidal ideation (yes/no) (Additional file 1: Table S9) neither for any of the significant secondary outcomes (Additional file 1: Table S10). The subgroup analysis of antidepressant use showed a significantly higher level of IL-4 among patients taking antidepressants as compared to patients who did not (*p* = 0.010), which was not significant after adjustment for multiple testing, whereas there were no differences for any of the primary or other significant secondary outcomes. Likewise, there were no impact of smoking status nor use of antipsychotics (Additional file 1: Tables S11 and S12). Furthermore, neither accounting for the time from sample collection to freezing, duration of storage nor blood contamination altered the results of primary outcomes (Additional file 1: Table S13).

## Discussion

This large case–control study compared CSF cytokine levels among 106 patients with recent-onset first-episode depression to 106 individually (age and sex) matched healthy controls. We did not find significant differences in CSF levels of the co-primary outcome cytokines, IL-6 and IL-8, whereas IL-4 levels were 40%, MCP-1 levels were 25% and MIP-1β levels were 16% higher among patients with depression compared to controls. However, only IL-4 levels were significantly elevated after adjustment for multiple testing.

IL-4, MCP-1 and MIP-1β have only been investigated in few prior studies in the context of depression. IL-4 is a signature cytokine of a Th2-response [30] with a known pro-inflammatory effect on macrophages [31]. However, IL-4 has been suggested to yield anti-inflammatory properties in the activation of microglia within a very complex system [32]. MCP-1 is a key chemokine involved in the regulation of monocyte/macrophage infiltration [33] and has a potential role in fatigue [34]. MIP-1β is also named CC chemokine ligand 4 (CCL4) and is essential to the regulation and modulation of inflammation, including T-cell chemotaxis [35]. To our knowledge, CSF IL-4 levels have not previously been compared between patients with depression and healthy controls. CSF levels of MIP-1β and MCP-1 have been reported lower among 15 patients with depression who had recently attempted suicide compared to 43 healthy controls [36], whereas a small study investigating CSF MCP-1 levels among 11 patients with depression compared to 27 healthy controls found no significant difference, however, this study was likely underpowered to identify contrasts [37]. Another small study compared 44 patients with depression to 21 patients with neurological symptoms and found no significant group differences regarding CSF levels of IL-4, MCP-1 and MIP-1β [38], whereas a study found higher CSF MCP-1 levels to be associated with more severe depressive symptoms in patients with Parkinson’s disease [39]. Regarding peripheral blood levels, meta-analyses found lower levels of serum IL-4 in patients with depression [4] and lower levels of serum MIP-1β in patients with depression [40] compared to controls. The network of cytokines is very complex, and the alterations most likely differ between cerebrospinal fluid and blood. Thus, the finding of increased levels of CSF IL-4 among patients with recent-onset depression could perhaps reflect an early protective mechanism against the pro-inflammatory alterations seen in the blood. However, studies comparative to ours exploring CSF IL-4 in other subgroups of patients with depression, e.g., patients with treatment resistant depression or recurrent depression could elucidate this theory further.

In contrast to a previous meta-analysis [8], we found no differences in the levels of the primary outcome cytokine, IL-8. However, a more recent meta-analysis did not either find significant differences in IL-8 levels between groups [9]. Since IL-8 was quantifiable in all our samples, our data support that IL-8 does not seem to have a significant role in the neuroinflammatory response of recent-onset depression. As the main function of IL-8 is recruitment and diapedesis of neutrophils [41] and as we previously revealed a lower neutrophil proportion in the CSF from the same population of patients with depression compared to healthy controls [7], it appears plausible that IL-8 is not a main cytokine related to depression.

That we found no significant difference in levels of the key pro-inflammatory, pleiotropic cytokine IL-6 [42, 43] is in contrast to prior evidence from meta-analyses revealing levels of key pro-inflammatory cytokines (including IL-6) to be higher in blood [4] and CSF [8, 9] from patients with depression compared to healthy controls. However, our study was designed to minimize confounding, and we excluded patients who had co-morbid conditions with potential substantial impact on the immune system, as opposed to most previous studies. Furthermore, baseline levels of (serum) IL-6 appear to be increasing with age [44], and the participants of our study were relatively young. Due to these factors, it is possible that the inflammatory response in the patients of our study was more difficult to detect than in previous studies, thus, reducing our ability to detect contrasts in IL-6 levels between groups and explaining some of the differences between results of our and previous studies. Furthermore, in our study only patients with recent-onset depression who had ongoing depression symptoms were included, also in contrast to most previous studies on CSF cytokine levels.

### Strengths and limitations

This study is strengthened by the large sample size with individual matching of patients and controls. It is also a considerable strength that patients are rather homogeneous, as all had recent-onset first-episode depression. That only patients with recent-onset first-episode depression were included, is a strength as our findings can contribute to the knowledge of the early phase of depression; thus prior to eventual treatment resistance and/or chronicity. Furthermore, we consider it strengths that we had a broad measurement of cytokines, that we provide information on the number of measurements below the LLOD and the LLOQ, especially as this could be important to future meta-analyses, and that we account for measurements below the LLOD and the LLOQ in the statistical analyses by using the censored log-normal model.

In the present study, measuring cytokine levels by multiplex panels, levels of CSF IL-6 were above LLOD in all samples, but only above LLOQ in samples from 13 (12.2%) patients and 12 (11.3%) healthy controls, and the IL-6 findings reported here should therefore be interpreted with caution. Furthermore, measurements of IL-4 were above LLOD in 93 (87.7%) patients and 56 (52.8%) controls, and therefore it was not possible to adjust IL-4 results for plate number, which could impact results as patients and healthy controls were not randomly distributed between the plates. The lack of random distribution between plates is a limitation of our study. Across all cytokines, only ten were above LLOD in more than 90% of samples (IL-6, IL-8, IL-15, IL-16, ICAM-1, IP-10, MCP-1, MIP-1β, IL-12 and IL-7), and only five were above LLOQ in more than 90% samples (IL-8, IL-15, ICAM-1, IP-10 and MCP-1). Our statistical analyses and ability to detect possible contrasts in inflammation between patients and controls are thus limited by the current boundaries of biomarker detection and quantification when using multiplex panels for CSF analyses. However, the multiplex kit provided by MesoScale is among the most sensitive multiplex kits currently available on the market and analyses of intra-assay variation revealed good reliability. It is difficult to evaluate the impact that these methodological limitations might have had on previous studies as most do not provide information on thresholds for LLOD and LLOQ, the number of samples below these thresholds or how samples below thresholds are handled in the statistical analyses, and future papers should provide this information. Furthermore, our results of increased MCP-1 and MIP-1β levels should be interpreted with caution as correction for multiple testing rendered MCP-1 and MIP-1β not statistically significant. Adding to this, the mean time from sample collection (first droplet of CSF) to freezing was 75.9 (15.9) minutes, and it is a limitation that cytokines excreted during this period are also included in the analyses.

As the immunological activation, and thus the cytokine response, also has a temporal aspect, future studies could include longitudinal explorations, e.g., follow-up of patients with recent-onset depression one or two years after first episode could provide valuable insight to the cytokine alterations within the CNS over the course of the depression. Furthermore, within recent years it has been suggested that inflammation as related to depression has a particular importance in the subgroup of patients with immune metabolic dysregulations and depression [45]. Thus, it could be considered a limitation to our study that patients and healthy controls were all selected to be somatically healthy and future studies could consider studying neuroimmunological alterations of CSF in a group of patients with depression and immune metabolic dysregulation. Simultaneously, standardization and development of multiplex kits for evaluation of CSF are of utmost importance, when investigating the probably minor, however, still potentially significant players in the neuroinflammatory response of depression.

## Conclusions

In this large study, including 106 patients with recent-onset first-episode depression and 106 individually matched (sex and age) healthy controls, we found no significant differences between groups in CSF levels of IL-6 and IL-8. IL-4, MCP-1 and MIP-1β were higher in CSF from patients compared to controls, and could potentially be important to depression, although only IL-4 survived correction for multiple testing. However, due to methodological challenges the results should be interpreted with caution. Future studies could consider exploring the neuroinflammatory response related to later stages of depression, alterations over time, and whether a more pronounced response is seen in other subgroups of patients with depression.

## Supplementary Information

**Additional file 1:** **Table S1.** Minor somatic co-morbidity in population. **Table S2.** Intra- and interassay variation. **Table S3.** LLOD and LLOQ. **Table S4.** Definitions of cytokine categories. **Table S5.** Cytokines above LLOQ. **Table S6.** Measurements above LLOD and LLOQ. **Table S7.** Summary of observed data. **Table S8.** CSF cytokines from patients with depression and healthy controls categorized by inflammatory properties. **Table S9.** Subgroup analysis of HDRS-17 score, HARS score and suicidal ideation for primary outcomes. **Table S10.** Subgroup analysis of HDRS-17 score, HARS score and suicidal ideation for secondary outcomes. **Table S11.** Subgroup analysis based on smoking status, antidepressants, and antipsychotics for primary outcomes. **Table S12.** Subgroup analysis based on smoking status, antidepressants, and antipsychotics for secondary outcomes. **Table S13.** Sensitivity analyses of primary outcomes. **Figure S1.** Participant distribution on plates. **Figure S2.** Percentages of samples with detectable and quantifiable levels of cytokines/chemokines illustrated pr. Plate. **Figure S3.** The distribution of concentrations of all outcome measures estimated from censored log-normal models related to the lower limit of detection (LLOD) and lower limit of quantification (LLOQ). Methods S1.

## Data Availability

The individual level data in the current study are not publicly available due to restrictions by the Danish data authorities but are available from the corresponding author (prof. Benros) on reasonable request that can be approved by the Danish data authorities. Some of the demographic data only have previously been published elsewhere [7].

## Abbreviations

BACS: Brief Assessment of Cognition in Schizophrenia
BBB: Blood–brain barrier
CI: Confidence interval
CSF: Cerebrospinal fluid
GAF: Global Assessment of Functioning
HARS: Hamilton Anxiety Rating Scale
HDRS-17: 17-Item Hamilton Depression Rating Scale
ICAM: Intercellular adhesion molecule
ICD-10: International Classification of Diseases 10th Revision
IL: Interleukin
INF: Interferon
IP-10: Interferon gamma-induced protein-10
LLOD: Lower limit of detection
LLOQ: Lower limit of quantification
MADRS-10: 10-Item Montgomery–Asberg Depression Rating Scale
MCP: Monocyte chemoattractant protein
MD: Relative mean difference
MDC: Macrophage-derived chemokine
MIP: Macrophage inflammatory protein
MMSE: Mini-Mental State Examination
MoCA: Montreal Cognitive Assessment
MSD: MesoScale
NSS: Neurological Soft Signs
PANNS: Positive and Negative Symptom Scale
SCAN: WHO Schedules for Clinical Assessment in Neuropsychiatry
TARC: Thymus- and activation-regulated chemokine
TNF: Tumor necrosis factor
