# Supplementary material for: Comparisons of 25 cerebrospinal fluid cytokines in a case–control study of 106 patients with recent-onset depression and 106 individually matched healthy subjects
Source: J Neuroinflammation. 2023 Apr 4;20:90. doi: 10.1186/s12974-023-02757-2 (PMC10071627; doi:10.1186/s12974-023-02757-2)
Supplement: Supplementary file 1 — Additional file 1: Table S1. Minor somatic co-morbidity in population. Table S2. Intra- and interassay variation. Table S3. LLOD and LLOQ. Table S4. Definitions of cytokine categories. Table S5. Cytokines above LLOQ. Table S6. Measurements above LLOD and LLOQ. Table S7. Summary of observed data. Table S8. CSF cytokines from patients with depression and healthy controls categorized by inflammatory properties. Table S9. Subgroup analysis of HDRS-17 score, HARS score and suicidal ideation for primary outcomes. Table S10. Subgroup analysis of HDRS-17 score, HARS score and suicidal ideation for secondary outcomes. Table S11. Subgroup analysis based on smoking status, antidepressants, and antipsychotics for primary outcomes. Table S12. Subgroup analysis based on smoking status, antidepressants, and antipsychotics for secondary outcomes. Table S13. Sensitivity analyses of primary outcomes. Figure S1. Participant distribution on plates. Figure S2. Percentages of samples with detectable and quantifiable levels of cytokines/chemokines illustrated pr. Plate. Figure S3. The distribution of concentrations of all outcome measures estimated from censored log-normal models related to the lower limit of detection (LLOD) and lower limit of quantification (LLOQ). Methods S1. [file 12974_2023_2757_MOESM1_ESM.docx]

**Comparisons of 25 cerebrospinal fluid cytokines in a case control study of 106 patients with recent-onset depression and 106 individually matched healthy controls**

Additional file

Content

[**Table S1 ǀ** Minor somatic co-morbidity in population 2](#_Toc108431684)

[**Table S2 ǀ** Intra- and interassay variation 3](#_Toc108431684)

[**Table S3 ǀ** LLOD and LLOQ 4](#_Toc108431685)

[**Table S4 ǀ** Definitions of cytokine categories 5](#_Toc108431686)

[**Table S5** ǀ Cytokines above LLOQ 6](#_Toc108431688)

[**Table S6 ǀ** Measurements above LLOD and LLOQ 7](#_Toc108431689)

[**Table S7 ǀ** Summary of observed data 8](#_Toc108431690)

[**Table S8 ǀ** CSF cytokines from patients with depression and healthy controls categorized by inflammatory properties 10](#_Toc108431691)

[**Table S9 ǀ** Subgroup analysis of HDRS-17 score, HARS score and suicidal ideation for primary outcomes 10](#_Toc108431691)

[**Table S10 ǀ** Subgroup analysis of HDRS-17 score, HARS score and suicidal ideation for secondary outcomes 11](#_Toc108431691)

**Table S11 ǀ** Subgroup analysis based on smoking status, antidepressants, and antipsychotics for primary outcomes 12

[**Table S12 ǀ** Subgroup analysis based on smoking status, antidepressants, and antipsychotics for secondary outcomes 13](#_Toc108431691)

[**Table S13 ǀ** Sensitivity analyses of primary outcomes 14](#_Toc108431692)

[**Figure S1.** Participant distribution on plates. 15](#_Toc108431693)

[**Figure S2.** Percentages of samples with detectable and quantifiable levels of cytokines/chemokines illustrated pr. plate. 16](#_Toc108431694)

[**Figure S3.** The distribution of concentrations of all outcome measures estimated from censored log-normal models related to the lower limit of detection (LLOD) and lower limit of quantification (LLOQ). 17](#_Toc108431695)

**e[Methods](#_Toc108431696)** [18](#_Toc108431696)

[**References** 21](#_Toc108431696)

| **Table S1 ǀ** Minor somatic co-morbidity in population | | | |
| --- | --- | --- | --- |
|  | Depression  (N = 106) | Healthy  (N = 106) | *p* value |
| **Any somatic co-morbidity**^a^ |  |  | 0.531 |
| Yes (%) | 59 (56.2) | 55 (51.9) |  |
| No (%) | 46 (43.8) | 51 (48.1) |  |
|  |  |  |  |
| **Minor somatic co-morbidity** |  |  | 0.182 |
| Any (%) | 25 (23.6) | 21 (19.8) |  |
| Skin disorders |  |  |  |
| Acne (%) | 1 (0.9) | 2 (1.9) |  |
| Atopica (%) | 2 (1.9) | 0 (0.0) |  |
| Psoriasis (%) | 2 (1.9) | 2 (1.9) |  |
| Other (%) | 1 (0.9) | 0 (0.0) |  |
| Headache |  |  |  |
| Migraine (%) | 4 (3.8) | 1 (0.9) |  |
| Other (%) | 3 (2.8) | 1 (0.9) |  |
| Musculoskeletal disorders (%) | 6 (5.7) | 7 (6.6) |  |
| Lactose intolerance (%) | 1 (0.9) | 6 (5.7) |  |
| Other (%) | 5 (4.7) | 1 (0.9) |  |
| None (%) | 81 (76.4) | 86 (81.1) |  |
|  |  |  |  |
| **Allergy^b^** |  |  |  |
| Medical (%) | 2 (1.9) | 0 (0.0) | 0.155 |
| Non-medical |  |  | 0.522 |
| Food (%) | 1 (1.0) | 4 (3.8) |  |
| Pollen (%) | 11 (10.6) | 13 (12.3) |  |
| ≥ 2 types (%) | 14 (13.5) | 13 (12.3) |  |
| Other (%) | 5 (4.8) | 5 (4.7) |  |
| None (%) | 71 (68.3) | 71 (67.0) |  |
|  |  |  |  |
| **Asthma** |  |  | 0.066 |
| Current (%) | 15 (14.2) | 5 (4.7) |  |
| Former (%) | 5 (4.7) | 5 (4.7) |  |
| None (%) | 86 (81.1) | 96 (90.6) |  |
|  |  |  |  |
| Participants were excluded if their condition were not considered mild or as for allergy, if they had had a daily intake of anti-allergy medication within the prior two weeks.  Categorical variables are shown in absolute numbers and (%). *p*-values are based on Pearson’s Chi-square test.  ^a^Data of one patient is missing. ^b^Data are missing for two patients and uncategorized for two other patients. | | | |

| **Table S2 ǀ** Intra- and interassay variation | | |
| --- | --- | --- |
|  | Intraassay variation  (CV%) | Interassay variation  (CV%) |
|  |  |  |
| IL-6 | 4.5 | 4.0 |
| IL-8 | 2.6 | 3.3 |
|  |  |  |
| IFN-γ | 3.0 | 3.8 |
| IL-1α | 5.4 | 8.5 |
| IL-1β | 3.0 | 3.1 |
| IL-2 | 3.4 | 5.1 |
| IL-4 | 4.5 | 5.7 |
| IL-5 | 7.4 | 13.5 |
| IL-7 | 2.7 | 6.8 |
| IL-10 | 2.8 | 5.2 |
| IL-12^a^ | 2.4 | 7.9 |
| IL-13 | 1.6 | 5.1 |
| IL-15 | 4.9 | 7.5 |
| IL-16 | 5.5 | 5.8 |
| IL-17A | 2.5 | 6.6 |
| IP-10 | 4.9 | 20.1 |
| MCP-1 | 4.9 | 11.2 |
| MCP-4 | 3.2 | 23.0 |
| MDC | 2.7 | 24.5 |
| MIP-1α | 4.1 | 8.5 |
| MIP-1β | 5.3 | 4.7 |
| TARC | 11.2 | 11.6 |
| TNF-α | 14.3 | 3.8 |
| TNF-β | 3.9 | 5.1 |
| ICAM-1 | 8.7 | 16.8 |
|  |  |  |
| ^a^IL-23p40  **Abbreviations**: CV: coefficient of variation. ICAM: intercellular adhesion molecule. IFN: interferon. IP: interferon gamma-induced protein-10. IL: interleukin. MCP: monocyte chemoattractant protein. MDC: macrophage-derived chemokine. MIP: macrophage inflammatory protein. TARC: thymus- and activation-regulated chemokine. TNF: tumor necrosis factor. | | |

| **Table S3 ǀ** LLOD and LLOQ | | | |
| --- | --- | --- | --- |
|  | LLOD^a^  (SSI) | LLOD^b^  (MesoScale) | LLOQ^b^  (MesoScale) |
|  |  |  |  |
| IL-6 | 0.05 | 0.06 | 0.633 |
| IL-8 | 0.02 | 0.07 | 0.591 |
|  |  |  |  |
| IFN-γ | 0.11 | 0.37 | 1.76 |
| IL-1α | 0.21 | 0.09 | 2.85 |
| IL-1β | 0.01 | 0.05 | 0.646 |
| IL-2 | 0.03 | 0.09 | 0.890 |
| IL-4 | 0.01 | 0.02 | 0.218 |
| IL-5 | 0.43 | 0.14 | 4.41 |
| IL-7 | 0.26 | 0.12 | 0.546 |
| IL-10 | 0.05 | 0.04 | 0.298 |
| IL-12^c^ | 1.32 | 0.33 | 1.32 |
| IL-13 | 1.65 | 0.24 | 4.21 |
| IL-15 | 0.28 | 0.15 | 0.774 |
| IL-16 | 1.21 | 2.83 | 19.1 |
| IL-17A | 1.64 | 0.31 | 3.19 |
| IP-10 | 0.7 | 0.37 | 1.37 |
| MCP-1 | 0.4 | 0.09 | 1.09 |
| MCP-4 | 30.1 | 1.69 | 5.13 |
| MDC | 3.8 | 1.22 | 88.3 |
| MIP-1α | 2.4 | 3.02 | 13.8 |
| MIP-1β | 0.4 | 0.17 | 1.02 |
| TARC | 1.6 | 0.22 | 3.32 |
| TNF-α | 0.27 | 0.04 | 0.690 |
| TNF-β | 0.24 | 0.08 | 0.465 |
| ICAM-1 | 43.9 | 1.94 | 15.0 |
|  |  |  |  |
| Concentrations are reported as pg/mL. Statistical analyses use LLOD from Statens Serum Institute and LLOQ provided by MesoScale.  ^a^As estimated by Statens Serum Institute. ^b^As provided by MesoScale.  ^c^IL-23p40  **Abbreviations**: CSF: cerebrospinal fluid. LLOD: lower limit of detection. LLOQ: lower limit of quantification. IFN: interferon. IP: interferon gamma-induced protein-10. IL: interleukin. MCP: monocyte chemoattractant protein. MDC: macrophage-derived chemokine. MIP: macrophage inflammatory protein. TARC: thymus- and activation-regulated chemokine. TNF: tumor necrosis factor. ICAM: intercellular adhesion molecule. | | | |

| **Table S4 ǀ** Definitions of cytokine categories | | |
| --- | --- | --- |
|  | | |
| Pro-inflammatory |  | IL-1ɑ(1), IL-1β(1), IL-6(1), IL-8(1), IL-17(1), INF-γ(1), TNF-ɑ(1), TNF-β(1) |
| Anti-inflammatory |  | IL-10(1), IL-12(1) |
|  |  |  |
| Innate^a^ |  | TNF-ɑ(2), TNF-β,^a^ IFN-γ(2), IL-1β(2), IL-4(2), IL-6(2), IL-10(2), IL-12(2), MIP-1β (CCL4)(2) |
| Adaptive |  | IL-2(1), IL-4(1), IL-5(1), IL-7(1), IL-13(1), IL-15(1) |
|  |  |  |
| Acute |  | IL-1ɑ(3), IL-1β(3), TNF-ɑ(3), TNF-β(3), IL-6(3), IL-8(3), IL-16(3), IL-17(3) |
| Chronic |  |  |
| Humoral |  | IL-4(3), IL-5(3), IL-7(3), IL-10(3), IL-13(3) |
| Cellular |  | IL-2(3), IL-12(3), IL-15(3), INF-γ(3) |
|  |  |  |
| T helper cell response |  |  |
| Th1-response |  | IL-12(4), INF-γ(4), IL-2(4) |
| Th2-response |  | IL-4(4), IL-5(4), IL-13(4) |
| Th17-response |  | IL-6(4), IL-17(4) |
| Treg |  | IL-10(4) |
|  |  |  |
| Only cytokines measured in the present study are listed.  ^a^The paper defines the innate response as “TNF” that here is interpreted as TNF-ɑ and TNF-β.  **Abbreviations**: IL: interleukin. INF: interferon. MIP: macrophage inflammatory protein. TNF: tumor necrosis factor.  1. Turner MD, Nedjai B, Hurst T, Pennington DJ. Cytokines and chemokines: At the crossroads of cell signalling and inflammatory disease. Vol. 1843, Biochimica et biophysica acta. Netherlands; 2014. p. 2563–82.  2. Lacy P, Stow JL. Cytokine release from innate immune cells: association with diverse membrane trafficking pathways. Blood. 2011 Jul;118(1):9–18.  3. Feghali CA, Wright TM. Cytokines in acute and chronic inflammation. Front Biosci. 1997 Jan;2:d12-26.  4. Himmerich H, Patsalos O, Lichtblau N, Ibrahim MAA, Dalton B. Cytokine Research in Depression: Principles, Challenges, and Open Questions. Front psychiatry. 2019;10:30. | | |

| **Table S5** ǀ Cytokines above LLOQ measured in CSF from 106 patients with depression and 106 individually matched healthy controls | | | | | | | |
| --- | --- | --- | --- | --- | --- | --- | --- |
|  |  | Depression (N=106) | | Healthy Controls (N>106) | | Relative mean difference  (95% CI) | *p*-values |
|  | Unit | N>  LLOQ | Mean  (95% CI) | N>  LLOQ | Mean  (95% CI) |  |  |
| IL-6^a,b^ | 10^-12^ g/mL | 13 | 0.85 (0.64 - 1.13) | 12 | 0.81 (0.61 - 1.10) | 1.04 (0.83 - 1.31) | 0.736 |
| IL-8 | 10^-12^ g/mL | 106 | 17.69 (16.71 - 18.72) | 106 | 20.40 (19.27-21.59) | 1.05 (0.96 - 1.16) | 0.294 |
|  |  |  |  |  |  |  |  |
| IFN-γ^a,b^ | 10^-12^ g/mL | 0 | NA | 0 | NA | NA | NA |
| IL-1α^a,b^ | 10^-14^ g/mL | 4 | 12.54 (1.12 - 140.57) | 2 | 8.01 (0.52 - 124.49) | 1.57 (0.45 - 5.50) | 0.485 |
| IL-1β^a,b^ | 10^-14^ g/mL | 0 | NA | 0 | NA | NA | NA |
| IL-2^a,b^ | 10^-14^ g/mL | 0 | NA | 0 | NA | NA | NA |
| IL-4^a,b^ | 10^-14^ g/mL | 0 | NA | 0 | NA | NA | NA |
| IL-5^a,b^ | 10^-12^ g/mL | 2 | 0.76 (0.15 - 3.75) | 5 | 1.13 (0.31 - 4.13) | 0.67 (0.32 - 1.42) | 0.297 |
| IL-7^a^ | 10^-12^ g/mL | 5 | 0.01 (0.00 - 0.19) | 7 | 0.03 (0.00 - 0.28) | 0.33 (0.05 - 2.02) | 0.231 |
| IL-10^a,b^ | 10^-14^ g/mL | 1 | NA | 0 | NA | NA | NA |
| IL-12^a,d^ | 10^-12^ g/mL | 44 | 4.21 (3.47 - 5.11) | 31 | 3.68 (2.97 - 4.57) | 1.07 (0.79 - 1.46) | 0.657 |
| IL-13^a,b^ | 10^-12^ g/mL | 0 | NA | 0 | NA | NA | NA |
| IL-15^a^ | 10^-12^ g/mL | 106 | 3.13 (2.91 - 3.36) | 105 | 3.24 (3.01 - 3.49) | 0.95 (0.84 - 1.08) | 0.444 |
| IL-16^a,b^ | 10^-12^ g/mL | 5 | 4.08 (2.11 - 7.88) | 21 | 8.65 (5.83 - 12.85) | 0.47 (0.29 - 0.78) | ***0.003***^c^ |
| IL-17A^a,b^ | 10^-13^ g/mL | 1 | 0.09 (0.00 - 23.55) | 5 | 0.81 (0.02 - 38.45) | 0.11 (0.01 - 1.92) | 0.130 |
| IP-10^a^ | 10^-11^ g/mL | 106 | 9.08 (8.08 - 10.19) | 105 | 9.22 (8.21 - 10.35) | 1.13 (0.93 - 1.38) | 0.230 |
| MCP-1^a^ | 10^-10^ g/mL | 106 | 3.38 (3.11 - 3.68) | 105 | 3.15 (2.90 - 3.43) | 1.22 (1.05-1.40) | ***0.008***^e^ |
| MCP-4^a,b^ | 10^-11^ g/mL | 5 | 0.00 (0.00 - 0.10) | 2 | 0.00 (0.00 - 0.06) | 5.78 (0.23 - 143.55) | 0.285 |
| MDC^a,b^ | 10^-12^ g/mL | 0 | NA | 1 | NA | NA | NA |
| MIP-1α^a,b^ | 10^-12^ g/mL | 0 | NA | 3 | NA | NA | NA |
| MIP-1β^a,b^ | 10^-12^ g/mL | 0 | NA | 3 | NA | NA | NA |
| TARC^a^ | 10^-12^ g/mL | 26 | 2.16 (1.78 - 2.62) | 32 | 2.48 (2.08 - 2.94) | 1.05 (0.83 - 1.33) | 0.710 |
| TNF-α^a^ | 10^-13^ g/mL | 10 | 1.50 (0.77 - 2.93) | 15 | 2.13 (1.20 - 3.79) | 0.70 (0.43 - 1.16) | 0.166 |
| TNF-β^a^ | 10^-14^ g/mL | 1 | 0.02 (0.00 - 23.59) | 5 | 0.30 (0.00 - 39.74) | 0.07 (0.00-2.40) | 0.139 |
| ICAM-1^a,b^ | 10^-9^  g/mL | 106 | 1.48 (1.40 - 1.57) | 106 | 1.41 (1.33 - 1.50) | 1.04 (0.94-1.16) | 0.401 |
| Estimated means, relative mean differences, CIs and *p*-values are based on censored log-normal models adjusted for sex, age and plate. ^a^Analyses included censored measurements. ^b^Not adjusted for plate due to too few measurements above LLOQ. ^c^*p*-value after correction for multiple testing: 0.046. ^d^IL-23p40. ^e^Did not survive correction for multiple testing.  **Abbreviations**: CI: confidence interval. CSF: cerebrospinal fluid. LLOQ: lower limit of quantification. IFN: interferon. IP: interferon gamma-induced protein-10. IL: interleukin. MCP: monocyte chemoattractant protein. MDC: macrophage-derived chemokine. MIP: macrophage inflammatory protein. TARC: thymus- and activation-regulated chemokine. TNF: tumor necrosis factor. sICAM: soluble intercellular adhesion molecule. | | | | | | | |

| **Table S6 ǀ** Measurements above LLOD and LLOQ | | | | | | | |
| --- | --- | --- | --- | --- | --- | --- | --- |
|  | **LLOD^a^** | | |  | **LLOQ^b^** | | |
|  | N total  (%)  > LLOD | N patients (%)  > LLOD | N controls (%)  > LLOD |  | N total  (%)  > LLOQ | N patients (%)  > LLOD | N controls (%)  > LLOQ |
|  | | | | | | | |
| IL-6 | 212 (100.0) | 106 (100.0) | 106 (100.0) |  | 25 (11.8) | 13 (12.2) | 12 (11.3) |
| IL-8 | 212 (100.0) | 106 (100.0) | 106 (100.0) |  | 212 (100.0) | 106 (100.0) | 106 (100.0) |
|  |  |  |  |  |  |  |  |
| IL-15 | 212 (100.0) | 106 (100.0) | 106 (100.0) |  | 211 (99.5) | 106 (100.0) | 105 (99.1) |
| IL-16 | 212 (100.0) | 106 (100.0) | 106 (100.0) |  | 26 (12.3) | 5 (4.7) | 21 (19.8) |
| ICAM-1 | 212 (100.0) | 106 (100.0) | 106 (100.0) |  | 212 (100.0) | 106 (100.0) | 106 (100.0) |
| IP-10 | 211 (99.5) | 106 (100.0) | 106 (100.0) |  | 211 (99.5) | 106 (100.0) | 105 (99.1) |
| MCP-1 | 211 (99.5) | 106 (100.0) | 105 (99.1) |  | 211 (99.5) | 106 (100.0) | 105 (99.1) |
| MIP-1β | 211 (99.5) | 106 (100.0) | 105 (99.1) |  | 3 (1.4) | 0 (0.0) | 3 (2.8) |
| IL-12^c^ | 209 (98.6) | 104 (98.1) | 105 (99.1) |  | 75 (35.4) | 44 (41.5) | 31 (29.2) |
| IL-7 | 192 (90.6) | 93 (87.7) | 99 (93.4) |  | 12 (5.7) | 5 (4.7) | 7 (6.6) |
| MDC | 188 (88.7) | 95 (89.6) | 93 (87.7) |  | 1 (0.5) | 0 (0.0) | 1 (0.9) |
| IL-10 | 171 (80.7) | 94 (88.7) | 77 (72.6) |  | 1 (0.5) | 1 (0.9) | 0 (0.0) |
| IFN-γ | 159 (75.0) | 92 (86.8) | 67 (63.2) |  | 0 (0.0) | 0 (0.0) | 0 (0.0) |
| MIP-1α | 151 (71.2) | 69 (65.1) | 82 (77.4) |  | 3 (1.4) | 0 (0.0) | 3 (2.8) |
| IL-4 | 149 (70.3) | 93 (87.7) | 56 (52.8) |  | 0 (0.0) | 0 (0.0) | 0 (0.0) |
| IL-5 | 146 (68.9) | 72 (67.9) | 74 (69.8) |  | 7 (3.3) | 2 (1.9) | 5 (4.7) |
| TARC | 127 (59.9) | 61 (57.5) | 66 (62.3) |  | 58 (27.4) | 26 (24.5) | 32 (30.2) |
| TNF-α | 62 (29.2) | 27 (25.5) | 35 (33.0) |  | 25 (11.8) | 10 (9.4) | 15 (14.2) |
| IL-2 | 45 (21.2) | 30 (28.3) | 15 (14.2) |  | 0 (0.0) | 0 (0.0) | 0 (0.0) |
| IL-13 | 30 (14.2) | 12 (11.3) | 18 (17.0) |  | 0 (0.0) | 0 (0.0) | 0 (0.0) |
| IL-1β | 27 (12.7) | 18 (17.0) | 9 (8.5) |  | 0 (0.0) | 0 (0.0) | 0 (0.0) |
| IL-17A | 25 11.8) | 15 (14.2) | 10 (9.4) |  | 6 (2.8) | 1 (0.9) | 5 (4.7) |
| IL-1α | 23 (10.8) | 13 (12.3) | 10 (9.4) |  | 6 (2.8) | 4 (3.8) | 2 (1.9) |
| TNF-β | 16 (7.5) | 8 (7.5) | 8 (7.5) |  | 6 (2.8) | 1 (0.9) | 5 (4.7) |
| MCP-4 | 7 (3.3) | 5 (4.7) | 2 (1.9) |  | 7 (3.3) | 5 (4.7) | 2 (1.9) |
|  | | | | | | | |
| ^a^As estimated by Statens Serum Institute. ^b^As estimated by MesoScale. ^c^IL-23p40  **Abbreviations**: CSF: cerebrospinal fluid. LLOD: lower limit of detection. LLOQ: lower limit of quantification. IFN: interferon. IP: interferon gamma-induced protein-10. IL: interleukin. MCP: monocyte chemoattractant protein. MDC: macrophage-derived chemokine. MIP: macrophage inflammatory protein. TARC: thymus- and activation-regulated chemokine. TNF: tumor necrosis factor. sICAM: soluble intercellular adhesion molecule. | | | | | | | |

| **Table S7 ǀ** Summary of observed data | | | | | | | | | |
| --- | --- | --- | --- | --- | --- | --- | --- | --- | --- |
|  |  | Depression (N=106) | | | | Healthy controls (N=106) | | | |
|  | Unit | Median | Range | N >LLOD | N >LLOQ | Median | Range | N >LLOD | N >LLOQ |
|  |  |  |  |  |  |  |  |  |  |
| IL-6 | 10^-12^ g/mL | 0.88 | 0.32 - 5.20 | 106 | 13 | 0.77 | 0.19 - 3.17 | 106 | 12 |
| IL-8 | 10^-12^ g/mL | 17.81 | 7.22 - 63.72 | 106 | 106 | 19.18 | 8.36 - 67.80 | 106 | 106 |
|  |  |  |  |  |  |  |  |  |  |
| IFN-γ^a^ | 10^-12^ g/mL | 0.27 | <0.11 - 3.89 | 92 | 0 | 0.15 | <0.11 - 1.64 | 67 | 0 |
| IL-1α^a^ | 10^-14^ g/mL | <20.70 | <20.70 - 2903.11 | 13 | 4 | <20.70 | <20.70 - 664.13 | 9 | 2 |
| IL-1β^a^ | 10^-14^ g/mL | <0.95 | <0.95 - 34.91 | 18 | 0 | <0.95 | <0.95 - 403.19 | 8 | 1 |
| IL-2^a^ | 10^-14^ g/mL | 2.92 | <2.92 - 37.21 | 28 | 0 | <2.92 | <2.92 - 19.08 | 15 | 0 |
| IL-4^a^ | 10^-14^ g/mL | 1.51 | <1.03 - 4.97 | 69 | 0 | <1.03 | <1.03 - 5.15 | 40 | 0 |
| IL-5^a^ | 10^-12^ g/mL | 0.52 | <0.43 - 11.35 | 71 | 2 | 0.56 | <0.43 - 14.89 | 74 | 5 |
| IL-7^a^ | 10^-12^ g/mL | 0.59 | <0.26 - 12.63 | 93 | 5 | 0.63 | <0.26 - 25.28 | 99 | 7 |
| IL-10^a^ | 10^-14^ g/mL | 8.15 | <5.17 - 76.60 | 87 | 1 | 6.79 | <5.17 - 59.43 | 71 | 0 |
| IL-12^a,b^ | 10^-12^ g/mL | 4.87 | <1.32 - 36.50 | 104 | 44 | 4.35 | <1.32 - 62.32 | 105 | 31 |
| IL-13^a^ | 10^-12^ g/mL | <1.65 | <1.65 - 2.56 | 12 | 0 | <1.65 | <1.65 - 2.51 | 18 | 0 |
| IL-15 | 10^-12^ g/mL | 2.92 | 1.57 - 10.87 | 106 | 106 | 3.13 | 1.18 - 38.21 | 106 | 105 |
| IL-16 | 10^-12^ g/mL | 9.24 | 2.48 - 44.59 | 106 | 5 | 9.90 | 3.18 - 117.41 | 106 | 21 |
| IL-17A^a^ | 10^-13^ g/mL | <16.35 | <16.35 - 117.15 | 15 | 1 | <16.35 | <16.35 - 680.19 | 10 | 5 |
| IP-10^a^ | 10^-11^ g/mL | 9.44 | 1.29 - 294.79 | 106 | 106 | 9.49 | <0.07 - 40.18 | 105 | 105 |
| MCP-1^a^ | 10^-10^ g/mL | 3.42 | 1.56 - 7.67 | 106 | 106 | 3.21 | <0.00 - 7.21 | 105 | 105 |
| MCP-4^a^ | 10^-11^ g/mL | <3.01 | <3.01 - 4.47 | 5 | 5 | <3.01 | <3.01 - 4.59 | 2 | 2 |
| MDC^a^ | 10^-12^ g/mL | 7.55 | <3.80 - 35.30 | 94 | 0 | 7.74 | <3.80 - 111.42 | 92 | 1 |
| MIP-1α^a^ | 10^-12^ g/mL | 2.93 | <2.37 - 13.30 | 68 | 0 | 3.61 | <2.37 - 32.12 | 82 | 3 |
| MIP-1β^a^ | 10^-12^ g/mL | 9.82 | 3.22 - 21.01 | 106 | 0 | 8.86 | <0.37 - 31.95 | 105 | 3 |
| TARC^a^ | 10^-12^ g/mL | 2.01 | <1.64 - 7.56 | 59 | 26 | 2.19 | <1.64 - 11.21 | 65 | 32 |
| TNF-α^a^ | 10^-13^ g/mL | <2.69 | <2.69 - 18.05 | 26 | 10 | <2.69 | <2.69 - 45.07 | 34 | 15 |
| TNF-β^a^ | 10^-14^ g/mL | <24.45 | <24.45 - 309.85 | 7 | 1 | <24.45 | <24.45 - 1404.66 | 8 | 5 |
| ICAM-1 | 10^-9^  g/mL | 1.45 | 0.77 - 4.96 | 106 | 106 | 1.39 | 0.60 - 3.46 | 106 | 106 |
|  |  |  |  |  |  |  |  |  |  |
| ^a^Measurements are censored below LLOD. ^b^IL-23p40  **Abbreviations**: CSF: cerebrospinal fluid. LLOD: lower limit of detection. LLOQ: lower limit of quantification. IFN: interferon. IP: interferon gamma-induced protein-10. IL: interleukin. MCP: monocyte chemoattractant protein. MDC: macrophage-derived chemokine. MIP: macrophage inflammatory protein. TARC: thymus- and activation-regulated chemokine. TNF: tumor necrosis factor. sICAM: soluble intercellular adhesion molecule. | | | | | | | | | |

| **Table S8 ǀ** CSF cytokines from patients with depression and healthy controls categorized by inflammatory properties | | |
| --- | --- | --- |
| Category | Relative mean difference (95% CI) | *p*-values |
|  |  |  |
| Pro-inflammatory | 1.02 (0.68-1.52) | 0.933 |
| Anti-inflammatory | 1.01 (0.74-1.38) | 0.947 |
|  |  |  |
| Innate | 1.05 (0.73-1.51) | 0.777 |
| Adaptive | 0.91 (0.63-1.30) | 0.590 |
|  |  |  |
| Acute | 0.96 (0.65-1.43) | 0.856 |
| Chronic |  |  |
| Humoral | 0.95 (0.66-1.35) | 0.756 |
| Cellular | 0.96 (0.69-1.33) | 0.788 |
|  |  |  |
| T helper cell response | | |
| Th1-response | 0.98 (0.70-1.37) | 0.921 |
| Th2-response | 0.92 (0.63-1.34) | 0.675 |
| Th17-response | 1.09 (0.75-1.60) | 0.648 |
| T_reg_-response | 1.07 (0.77-1.48) | 0.700 |
|  |  |  |
| Analyses are based on censored log-normal models adjusted for sex, age and plate with standardized cytokines combined in categories with measurements censored below LLOD. Cytokine categories are described in Table S4.  **Abbreviations**: CI: confidence interval. CSF: cerebrospinal fluid. LLOD: lower limit of detection. LLOQ: lower limit of quantification. | | |

| **Table S9 ǀ** Subgroup analysis of HDRS-17 score, HARS score and suicidal ideation for primary outcomes | | | | | | |
| --- | --- | --- | --- | --- | --- | --- |
|  |  | N | Mean  (95% CI) | Relative mean difference to controls | *p*-value^a^ | Effect heterogeneity *p*-value^b^ |
| *Severity of depression* | |  |  |  |  |  |
| ***IL-6 pg/mL*** | |  |  |  |  |  |
|  | Patients | 106 |  |  |  |  |
|  | HDRS-17 score > 24 | 29 | 0.85 (0.70-1.02) | 1.03 (0.82-1.30) | 0.786 | 0.436 |
|  | HDRS-17 score ≤ 24 | 77 | 0.91 (0.82-1.03) | 1.13 (0.94-1.35) | 0.194 |  |
|  | Healthy controls (ref) | 106 | 0.81 (0.73-0.89) | 1.00 (ref) |  |  |
|  |  |  |  |  |  |  |
| ***IL-8 pg/mL*** | |  |  |  |  |  |
|  | Patients | 106 |  |  |  |  |
|  | HDRS-17 score > 24 | 29 | 18.09 (16.22-20.17) | 1.06 (0.93-1.21) | 0.382 | 0.877 |
|  | HDRS-17 score ≤ 24 | 77 | 17.54 (16.40-18.75) | 1.05 (0.95-1.17) | 0.359 |  |
|  | Healthy controls (ref) | 106 | 20.40 (19.26-21.59) | 1.00 (ref) |  |  |
|  |  |  |  |  |  |  |
| *Severity of co-morbid anxiety* | |  |  |  |  |  |
| ***IL-6 pg/mL*** | |  |  |  |  |  |
|  | Patients | 106 |  |  |  |  |
|  | HARS score > 20 | 58 | 0.91 (0.80-1.04) | 1.13 (0.93-1.36) | 0.224 | 0.584 |
|  | HARS score ≤ 20 | 48 | 0.88 (0.76-1.02) | 1.07 (0.87-1.30) | 0.529 |  |
|  | Healthy controls (ref) | 106 | 0.81 (0.73-0.89) | 1.00 (ref) |  |  |
|  |  |  |  |  |  |  |
| ***IL-8 pg/mL*** | |  |  |  |  |  |
|  | Patients | 106 |  |  |  |  |
|  | HARS score > 20 | 58 | 17.12 (15.85-18.50) | 1.04 (0.93-1.16) | 0.543 | 0.523 |
|  | HARS score ≤ 20 | 48 | 18.39 (16.89-20.02) | 1.07 (0.96-1.21) | 0.220 |  |
|  | Healthy controls (ref) | 106 | 20.40 (19.27-21.59) | 1.00 (ref) |  |  |
|  |  |  |  |  |  |  |
| *Suicidal ideation* | |  |  |  |  |  |
| ***IL-6 pg/mL*** | |  |  |  |  |  |
|  | Patients | 106 |  |  |  |  |
|  | Suicidal ideation yes | 40 | 0.93 (0.79-1.09) | 1.12 (0.91-1.38) | 0.283 | 0.739 |
|  | Suicidal ideation no | 66 | 0.88 (0.77-0.99) | 1.08 (0.90-1.30) | 0.399 |  |
|  | Healthy controls (ref) | 106 | 0.81 (0.73-0.89) | 1.00 (ref) |  |  |
|  |  |  |  |  |  |  |
| ***IL-8 pg/mL*** | |  |  |  |  |  |
| Patients | | 106 |  |  |  |  |
|  | Suicidal ideation yes | 40 | 18.10 (16.49-19.86) | 1.06 (0.94-1.19) | 0.369 | 0.927 |
|  | Suicidal ideation no | 66 | 17.44 (16.23-18.75) | 1.05 (0.94-1.17) | 0.363 |  |
|  | Healthy controls (ref) | 106 | 20.40 (19.26-21.59) | 1.00 (ref) |  |  |
|  | | | | | | |
| Subgroup analyses are similar to models of primary outcomes adjusted for sex, age and plate, and include the interaction between group and the subgroup variable. The analyses are based on levels above LLOD.  ^a^*p*-value for relative mean difference versus controls within subgroup. ^b^: *p*-value for effect heterogeneity based on test of interaction.  Abbreviations: HARS: Hamilton Anxiety Rating Scale. HDRS-17: 17-item Hamilton Depression Rating scale. IL: interleukin. | | | | | | |

| **Table S10 ǀ** Subgroup analysis of HDRS-17 score, HARS score and suicidal ideation for significant secondary outcomes | | | | | | |
| --- | --- | --- | --- | --- | --- | --- |
|  |  | N | Mean  (95% CI) | Relative mean difference to controls | *p*-value^a^ | Effect heterogeneity *p*-value^b^ |
| *Severity of depression* | |  |  |  |  |  |
| ***IL-4 pg/mL*** | |  |  |  |  |  |
|  | Patients | 106 |  |  |  |  |
|  | HDRS-17 score > 24 | 29 | 1.48 (1.17 - 1.86) | 1.17 (0.88 - 1.56) | 0.272 | 0.119 |
|  | HDRS-17 score ≤ 24 | 77 | 1.21 (1.05 - 1.41) | 0.95 (0.75 - 1.19) | 0.633 |  |
|  | Healthy controls (ref) | 106 | 0.68 (0.00 - >100) | 1.00 (ref) |  |  |
| ***MCP-1 pg/mL*** | |  |  |  |  |  |
|  | Patients | 106 |  |  |  |  |
|  | HDRS-17 score > 24 | 29 | 3.14 (2.61 - 3.78) | 1.16 (0.92 - 1.45) | 0.208 | 0.337 |
|  | HDRS-17 score ≤ 24 | 77 | 3.48 (3.11 - 3.90) | 1.29 (1.08 - 1.54) | ***0.006*** |  |
|  | Healthy controls (ref) | 106 | 3.12 (2.83 - 3.44) | 1.00 (ref) |  |  |
| ***MIP-1β pg/mL*** | |  |  |  |  |  |
|  | Patients | 106 |  |  |  |  |
|  | HDRS-17 score > 24 | 29 | 8.88 (7.67 - 10.29) | 1.08 (0.91 - 1.30) | 0.380 | 0.252 |
|  | HDRS-17 score ≤ 24 | 77 | 9.80 (8.96 - 10.73) | 1.20 (1.04 - 1.38) | 0.012 |  |
|  | Healthy controls (ref) | 106 | 9.00 (8.33 - 9.72) | 1.00 (ref) |  |  |
| *Severity of co-morbid anxiety* | |  |  |  |  |  |
| ***IL-4 pg/mL*** | |  |  |  |  |  |
|  | Patients | 106 |  |  |  |  |
|  | HARS score > 20 | 58 | 1.35 (1.15 - 1.59) | 1.02 (0.80 - 1.30) | 0.861 | 0.815 |
|  | HARS score ≤ 20 | 48 | 1.20 (0.99 - 1.45) | 0.99 (0.77 - 1.29) | 0.950 |  |
|  | Healthy controls (ref) | 106 | 0.68 (0.00 - >100) | 1.00 (ref) |  |  |
| ***MCP-1 pg/mL*** | |  |  |  |  |  |
|  | Patients | 106 |  |  |  |  |
|  | HARS score > 20 | 58 | 3.35 (2.93 - 3.82) | 1.25 (1.04 - 1.52) | ***0.020*** |  |
|  | HARS score ≤ 20 | 48 | 3.43 (2.96 - 3.96) | 1.24 (1.02 - 1.51) | ***0.006*** | 0.900 |
|  | Healthy controls (ref) | 106 | 3.12 (2.83 - 3.44) | 1.00 (ref) |  |  |
| ***MIP-1β pg/mL*** | |  |  |  |  |  |
|  | Patients | 106 |  |  |  |  |
|  | HARS score > 20 | 58 | 9.08 (8.18 - 10.08) | 1.11 (0.95 - 1.29) | 0.177 |  |
|  | HARS score ≤ 20 | 48 | 10.13 (9.03 - 11.35) | 1.23 (1.05 - 1.44) | ***0.009*** | 0.199 |
|  | Healthy controls (ref) | 106 | 9.00 (8.33 - 9.72) | 1.00 (ref) |  |  |
| *Suicidal ideation* | |  |  |  |  |  |
| ***IL-4 pg/mL*** | |  |  |  |  |  |
|  | Patients | 106 |  |  |  |  |
|  | Suicidal ideation yes | 40 | 1.34 (1.10 - 1.64) | 1.11 (0.85 - 1.44) | 0.455 |  |
|  | Suicidal ideation no | 66 | 1.25 (1.07 - 1.46) | 0.96 (0.76 - 1.21) | 0.701 | 0.249 |
|  | Healthy controls (ref) | 106 | 0.68 (0.00 - >100) | 1.00 (ref) |  |  |
| ***MCP-1 pg/mL*** | |  |  |  |  |  |
| Patients | | 106 |  |  |  |  |
|  | Suicidal ideation yes | 40 | 3.39 (2.89 - 3.97) | 1.23 (1.00 - 1.52) | ***0.046*** |  |
|  | Suicidal ideation no | 66 | 3.38 (2.99 - 3.82) | 1.25 (1.04 - 1.51) | ***0.016*** | 0.874 |
|  | Healthy controls (ref) | 106 | 3.12 (2.83 - 3.44) | 1.00 (ref) |  |  |
| ***MIP-1β pg/mL*** | |  |  |  |  |  |
|  | Suicidal ideation yes | 40 | 9.94 (8.77 - 11.27) | 1.20 (1.02 - 1.42) | ***0.027*** |  |
|  | Suicidal ideation no | 66 | 9.31 (8.44 - 10.27) | 1.14 (0.98 - 1.32) | 0.081 | 0.496 |
|  | Healthy controls (ref) | 106 | 9.00 (8.33 - 9.72) | 1.00 (ref) |  |  |
| Subgroup analyses are similar to models of primary outcomes adjusted for sex, age and plate, and include the interaction between group and the subgroup variable. The analyses are based on levels above LLOD.  ^a^*p*-value for relative mean difference versus controls within subgroup. ^b^: *p*-value for effect heterogeneity based on test of interaction.  Abbreviations: HARS: Hamilton Anxiety Rating Scale. HDRS-17: 17-item Hamilton Depression Rating scale. IL: interleukin. | | | | | | |

| **Table SS11 ǀ** Subgroup analysis based on smoking status, antidepressants, and antipsychotics for primary outcomes | | | | | | | | | | | | | | |  |
| --- | --- | --- | --- | --- | --- | --- | --- | --- | --- | --- | --- | --- | --- | --- | --- |
|  |  | | | N | | | Mean  (95% CI) | | Relative mean difference to controls | | *p*-value^a^ | | Effect heterogeneity *p*-value^b^ | |  |
| *Smoking status* | | | |  | | |  | | |  | |  | |  | |
| ***IL-6 pg/mL*** | | | |  | | |  | | |  | |  | |  | |
| Patients | | | 106 | | |  | | |  | |  | |  | |  |
| Smokers | | | 32 | | | 0.94 (0.79 - 1.13) | | | 1.15 (0.92 - 1.43) | | 0.219 | | 0.534 | |  |
| Non-smokers | | | 74 | | | 0.88 (0.78 - 0.98) | | | 1.07 (0.90 - 1.29) | | 0.440 | |  |  |  |
| Healthy controls (ref) | | | 106 | | | 0.81 (0.73 - 0.89) | | | 1.00 (ref) | |  | |  | |  |
|  | | |  | | |  | | |  | |  | |  | |  |
| ***IL-8 pg/mL*** | | | |  | | |  | | |  | |  | |  | |
| Patients | | | 106 | | |  | | |  | |  | |  | |  |
| Smokers | | | 32 | | | 18.59 (16.76 - 20.62) | | | 1.08 (0.95 - 1.23) | | 0.256 | | 0.600 | |  |
| Non-smokers | | | 74 | | | 17.31 (16.17 - 18.53) | | | 1.04 (0.94 - 1.16) | | 0.440 | |  |  |  |
| Healthy controls (ref) | | | 106 | | | 20.40 (19.27 - 21.59) | | | 1.00 (ref) | |  | |  | |  |
|  | | |  | | |  | | |  | |  | |  | |  |
| *Antidepressants use* | | | |  | | |  | | |  | |  | |  | |
| ***IL-6 pg/mL*** | | | |  | | |  | | |  | |  | |  | |
| Patients | | | 106 | | |  | | |  | |  | |  | |  |
| Antidepressants: yes | | | 35 | | | 1.02 (0.86 - 1.21) | | | 1.25 (1.00 - 1.55) | | ***0.048*** | | 0.075 | |  |
| Antidepressants: no | | | 71 | | | 0.84 (0.74 - 0.94) | | | 1.03 (0.86 - 1.23) | | 0.774 | |  |  |  |
| Healthy controls (ref) | | | 106 | | | 0.81 (0.73 - 0.89) | | | 1.00 (ref) | |  | |  | |  |
|  | | |  | | |  | | |  | |  | |  | |  |
| ***IL-8 pg/mL*** | | | |  | | |  | | |  | |  | |  | |
| Patients | | | 106 | | |  | | |  | |  | |  | |  |
| Antidepressants: yes | | | 35 | | | 17.29 (15.64 - 19.11) | | | 1.03 (0.91 - 1.17) | | 0.658 | | 0.575 | |  |
| Antidepressants: no | | | 71 | | | 17.89 (16.68 - 19.19) | | | 1.07 (0.96 - 1.19) | | 0.236 | |  | |  |
| Healthy controls (ref) | | | 106 | | | 20.39 (19.26 - 21.59) | | | 1.00 (ref) | |  | |  | |  |
|  | | |  | | |  | | |  | |  | |  | |  |
| *Antipsychotics use* | | | |  | | |  | | |  | |  | |  | |
| ***IL-6 pg/mL*** | | | |  | | |  | | |  | |  | |  | |
| Patients | | | 106 | | |  | | |  | |  | |  | |  |
| Antipsychotics: yes | | | 12 | | | 0.96 (0.72 - 1.29) | | | 1.15 (0.84 - 1.58) | | 0.390 | | 0.732 | |  |
| Antipsychotics: no | | | 94 | | | 0.89 (0.80 - 0.98) | | | 1.09 (0.92 - 1.29) | | 0.332 | |  | |  |
| Healthy controls (ref) | | | 106 | | | 0.81 (0.73 - 0.89) | | | 1.00 (ref) | |  | |  | |  |
|  | | |  | | |  | | |  | |  | |  | |  |
| ***IL-8 pg/mL*** | | | |  | | |  | | |  | |  | |  | |
| Patients | | | | 106 | | |  | | |  | |  | |  | |
| Antipsychotics: yes | | | 12 | | | 16.71 (14.09 - 19.82) | | | 0.96 (0.80 - 1.16) | | 0.683 | | 0.262 | |  |
| Antipsychotics: no | | | 94 | | | 17.82 (16.77 - 18.93) | | | 1.07 (0.97 - 1.18) | | 0.196 | |  | |  |
| Healthy controls (ref) | | | 106 | | | 20.39 (19.27 - 21.59) | | | 1.00 (ref) | |  | |  | |  |
|  | | | | | | | | | | | | | | |  |
| Subgroup analyses are similar to models of primary outcomes adjusted for sex, age and plate, and include the interaction between group and the subgroup variable. The analyses are based on levels above LLOD.  ^a^*p*-value for relative mean difference versus controls within subgroup. ^b^: *p*-value for effect heterogeneity based on test of interaction.  Abbreviations: HARS: Hamilton Anxiety Rating Scale. HDRS-17: 17-item Hamilton Depression Rating scale. IL: interleukin. | | | | | | | | | | | | | | |  |

| **Table S12 ǀ** Subgroup analysis based on smoking status, antidepressants, and antipsychotics for significant secondary outcomes | | | | | | |
| --- | --- | --- | --- | --- | --- | --- |
|  |  | N | Mean  (95% CI) | Relative mean difference to controls | *p*-value^a^ | Effect heterogeneity *p*-value^b^ |
| *Smoking status* | |  |  |  |  |  |
| ***IL-4 pg/mL*** | |  |  |  |  |  |
|  | Patients | 106 |  |  |  |  |
|  | Smokers | 32 | 1.28 (1.02 - 1.60) | 1.07 (0.81 - 1.42) | 0.644 | 0.535 |
|  | Non-smokers | 74 | 1.28 (1.10 - 1.49) | 0.98 (0.78 - 1.24) | 0.884 |  |
|  | Healthy controls (ref) | 106 | 0.68 (0.00 - >100) | 1.00 (ref) |  |  |
| ***MCP-1 pg/mL*** | |  |  |  |  |  |
|  | Patients | 106 |  |  |  |  |
|  | Smokers | 32 | 3.56 (2.98 - 4.25) | 1.28 (1.03 - 1.59) | ***0.029*** | 0.742 |
|  | Non-smokers | 74 | 3.31 (2.95 - 3.72) | 1.23 (1.03 - 1.47) | ***0.023*** |  |
|  | Healthy controls (ref) | 106 | 3.12 (2.83 - 3.44) | 1.00 (ref) |  |  |
| ***MIP-1β pg/mL*** | |  |  |  |  |  |
|  | Patients | 106 |  |  |  |  |
|  | Smokers | 32 | 8.96 (7.79 - 10.31) | 1.08 (0.91 - 1.28) | 0.385 | 0.199 |
|  | Non-smokers | 74 | 9.80 (8.94 - 10.75) | 1.21 (1.05 - 1.39) | ***0.010*** |  |
|  | Healthy controls (ref) | 106 | 9.00 (8.33 - 9.72) | 1.00 (ref) |  |  |
| *Antidepressants use* | |  |  |  |  |  |
| ***IL-4 pg/mL*** | |  |  |  |  |  |
|  | Patients | 106 |  |  |  |  |
|  | Antidepressants: yes | 35 | 1.58 (1.29 - 1.94) | 1.25 (0.96 - 1.63) | 0.101 | ***0.010*** |
|  | Antidepressants: no | 71 | 1.15 (0.98 - 1.34) | 0.89 (0.71 - 1.12) | 0.326 |  |
|  | Healthy controls (ref) | 106 | 0.69 (0.00 - >100) | 1.00 (ref) |  |  |
| ***MCP-1 pg/mL*** | |  |  |  |  |  |
|  | Patients | 106 |  |  |  |  |
|  | Antidepressants: yes | 35 | 3.41 (2.87 - 4.04) | 1.25 (1.01 - 1.55) | ***0.045*** |  |
|  | Antidepressants: no | 71 | 3.37 (2.99 - 3.80) | 1.25 (1.04 - 1.49) | ***0.018*** | 0.980 |
|  | Healthy controls (ref) | 106 | 3.12 (2.83 - 3.44) | 1.00 (ref) |  |  |
| ***MIP-1β pg/mL*** | |  |  |  |  |  |
|  | Patients | 106 |  |  |  |  |
|  | Antidepressants: yes | 35 | 9.36 (8.17 - 10.72) | 1.14 (0.96 - 1.36) | 0.124 |  |
|  | Antidepressants: no | 71 | 9.63 (8.76 - 10.59) | 1.17 (1.02 - 1.36) | ***0.030*** | 0.193 |
|  | Healthy controls (ref) | 106 | 9.00 (8.33 - 9.72) | 1.00 (ref) |  |  |
| *Antipsychotics use* | |  |  |  |  |  |
| ***IL-4 pg/mL*** | |  |  |  |  |  |
|  | Patients | 106 |  |  |  |  |
|  | Antipsychotics: yes | 12 | 1.40 (0.97 - 2.02) | 1.17 (0.78 - 1.76) | 0.441 |  |
|  | Antipsychotics: no | 94 | 1.27 (1.11 - 1.45) | 0.99 (0.79 - 1.23) | 0.905 | 0.390 |
|  | Healthy controls (ref) | 106 | 0.68 (0.00 - >100) | 1.00 (ref) |  |  |
| ***MCP-1 pg/mL*** | |  |  |  |  |  |
| Patients | | 106 |  |  |  |  |
|  | Antipsychotics: yes | 12 | 3.16 (2.36 - 4.23) | 1.14 (0.83 - 1.57) | 0.421 |  |
|  | Antipsychotics: no | 94 | 3.41 (3.08 - 3.78) | 1.26 (1.07 - 1.50) | ***0.007*** | 0.516 |
|  | Healthy controls (ref) | 106 | 3.12 (2.83 - 3.44) | 1.00 (ref) |  |  |
| ***MIP-1β pg/mL*** | |  |  |  |  |  |
|  | Antipsychotics: yes | 12 | 8.33 (6.61 - 10.50) | 1.01 (0.78 - 1.30) | 0.945 |  |
|  | Antipsychotics: no | 94 | 9.71 (8.95 - 10.54) | 1.19 (1.04 - 1.36) | ***0.012*** | 0.193 |
|  | Healthy controls (ref) | 106 | 8.99 (8.33 - 9.71) | 1.00 (ref) |  |  |
| Subgroup analyses are similar to models of primary outcomes adjusted for sex, age and plate, and include the interaction between group and the subgroup variable. The analyses are based on levels above LLOD.  ^a^*p*-value for relative mean difference versus controls within subgroup. ^b^: *p*-value for effect heterogeneity based on test of interaction.  Abbreviations: HARS: Hamilton Anxiety Rating Scale. HDRS-17: 17-item Hamilton Depression Rating scale. IL: interleukin. | | | | | | |

| **Table S13 ǀ** Sensitivity analyses of primary outcomes | | | |
| --- | --- | --- | --- |
|  | Mean relative difference | 95% CI | *p*-value |
| ***Model 1 (primary model): Adjusted for age, sex and plate*** | | | |
| IL-6 | 1.10 | (0.93-1.30) | 0.276 |
| IL-8 | 1.05 | (0.96-1.16) | 0.294 |
|  |  |  |  |
| ***Model 2: Adjusted for age, sex, plate and time from collection to freezing*** | | | |
| IL-6 | 1.09 | (0.92 - 1.29) | 0.309 |
| IL-8 | 1.07 | (0.97 - 1.17) | 0.209 |
|  |  |  |  |
| ***Model 3: Adjusted for age, sex, plate and duration of storage*** | | | |
| IL-6 | 1.10 | (0.93 - 1.30) | 0.278 |
| IL-8 | 1.05 | (0.96 - 1.16) | 0.285 |
|  |  |  |  |
| ***Model 4: Adjusted for age, sex, plate and blood contamination*** | | | |
| IL-6 | 1.10 | (0.93 - 1.30) | 0.275 |
| IL-8 | 1.04 | (0.94 - 1.15) | 0.407 |
|  |  |  |  |
| ***Model 4 (fully adjusted):***  ***Adjusted for age, sex, plate, time from collection to freezing, duration of storage and blood contamination*** | | | |
| IL-6 | 1.09 | (0.92 - 1.30) | 0.320 |
| IL-8 | 1.06 | (0.96 - 1.17) | 0.280 |
|  |  |  |  |
|  |  |  |  |
| All models are censored log-normal models with measurements below LLOD censored.  **Abbreviations:** CI: confidence interval. IL: interleukin. | | | |


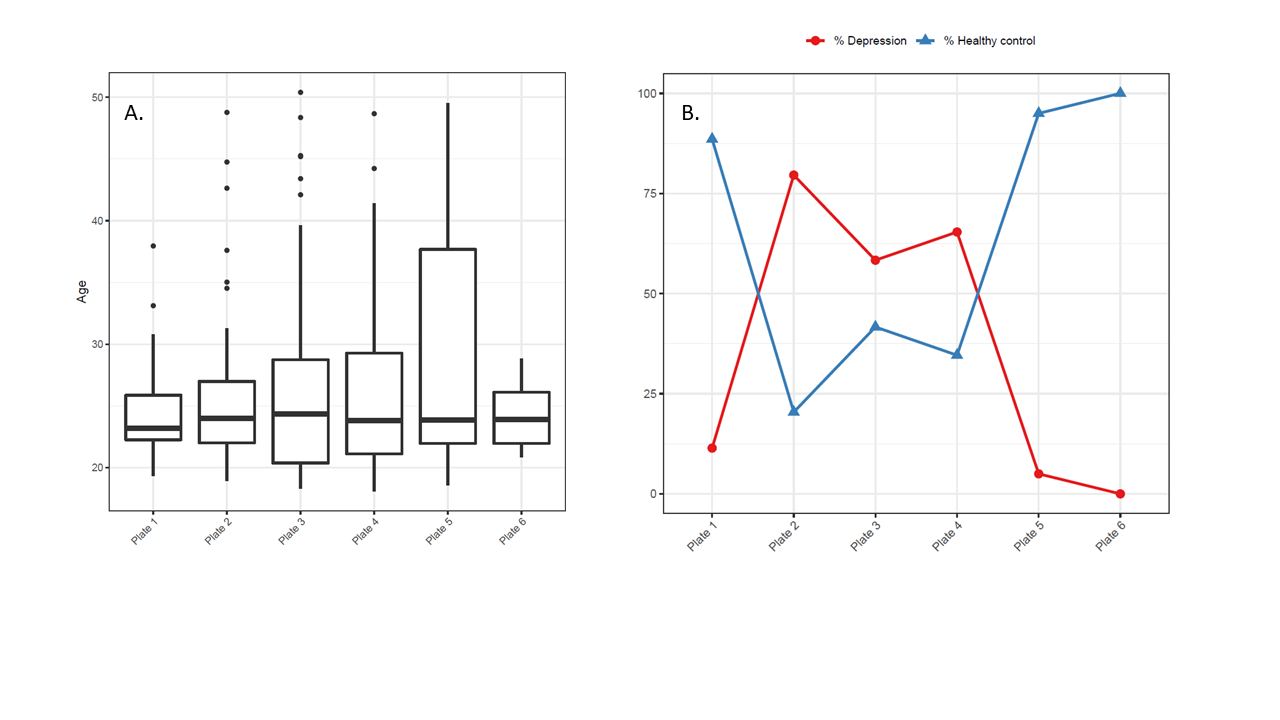


### **Figure S1.** Participant distribution on plates.

**A**. Illustrates the age distribution to the plates. **B**. Illustrates the distribution of patients and healthy controls to plates.


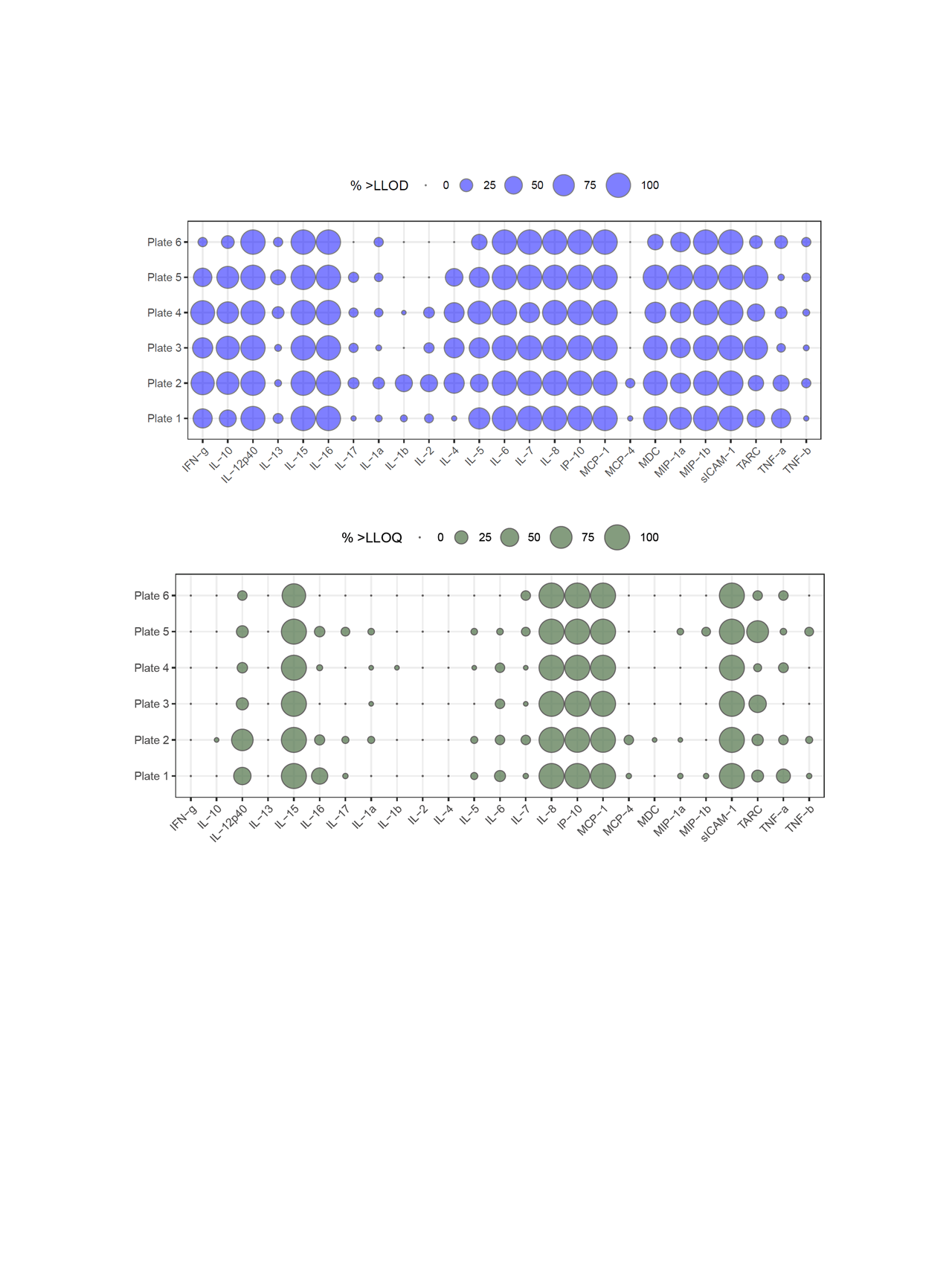


### **Figure S2.** Percentages of samples with detectable and quantifiable levels of cytokines/chemokines illustrated pr. plate.


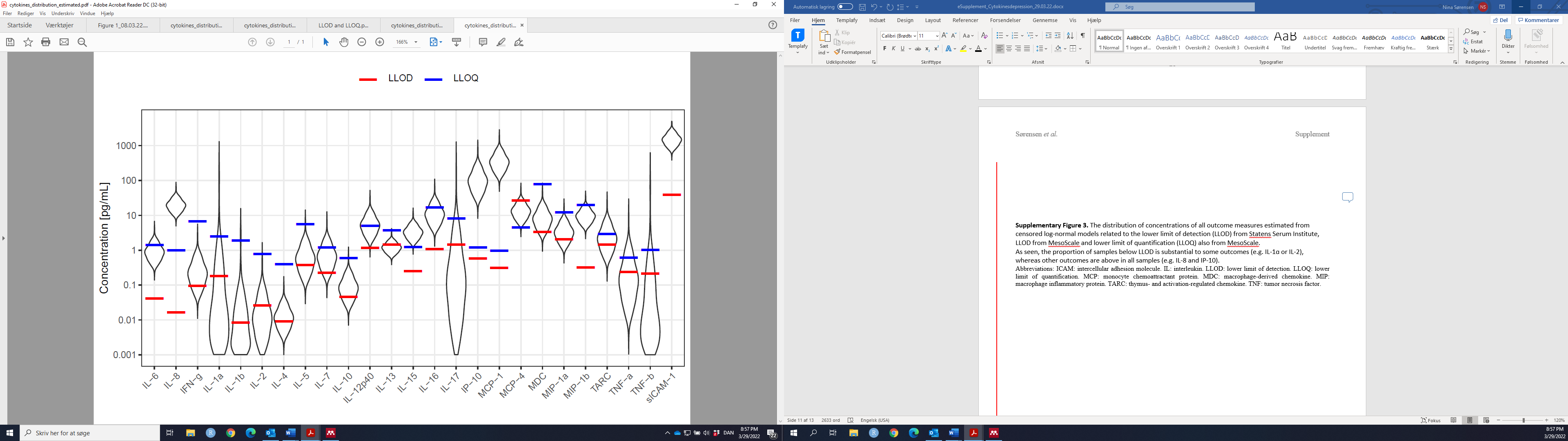


### **Figure S3.** The distribution of concentrations of all outcome measures estimated from censored log-normal models related to the lower limit of detection (LLOD) and lower limit of quantification (LLOQ).

LLOD is from Statens Serum Institute, LLOQ is from MesoScale. As seen, the proportion of samples below LLOD is substantial to some outcomes (e.g. IL-1ɑ or IL-2), whereas other outcomes are above in all samples (e.g. IL-8 and IP-10).

*Abbreviations:* ICAM: intercellular adhesion molecule. IL: interleukin. IP-10: Interferon gamma-induced protein-10. LLOD: lower limit of detection. LLOQ: lower limit of quantification. MCP: monocyte chemoattractant protein. MDC: macrophage-derived chemokine. MIP: macrophage inflammatory protein. TARC: thymus- and activation-regulated chemokine. TNF: tumor necrosis factor.

### **eMethods**

ICAM-1 was quantified by the Vascular Injury Panel 2 (human) kit (Mesoscale (MSD), Rockville, USA) following the manufacturer’s instruction. In brief, the samples were diluted 5-fold prior to analysis and 25 μL of diluted sample was added to the wells and incubated for two hours. After washing, 25 μL of detection antibody solution was added to each well and the plate incubated for one hour. The plates were washed again and 150 μL of 1X Read Buffer T was added to each well and the plate was read without further incubation. IL-1ɑ, IL-5, IL-7, IL12, IL-15, IL-16, IL-17A, and TNF-β were quantified by the Cytokine Panel 1 (human) kit (MSD), INF-γ, IL-1β, IL-2, IL-4, IL-6, IL-8, IL-10, IL-12p70, IL-13 and TNF-ɑ by the Proinflammatory Panel 1 (human) kit (MSD) and TARC, IP-10, MIP-1ɑ, IL-8, MCP-1, MDC and MCP-4 by the Chemokine Panel 1 (human) kit (MSD) following the manufacturer’s instruction. After washing of plates, 50 μL of sample (undiluted) was added to each well, sealed and incubated for two hours. After washing, 25 μL of detection antibody solution was added to each well and the plate incubated for two hours. The plates were washed again and 150 μL of 1X Read Buffer T was added to each well and the plate read without further incubation for Cytokine Panel 1, whereas 150 μL of 2X Read Buffer T was added to each well for the Chemokine Panel 1 and the Proinflammation Panel 1 10 minutes incubation was applied before reading the Chemokine Panel 1 plate, whereas the Proinflammatory Panel 1 plate was read immediately after adding read buffer. Final quantification from the calibrator curves on each plate using 4PL logistic regression was done with the MSD Workbench software.

*Quality control*

Inter-assay variation was determined by a standard curve and low/high controls in duplicates (N=5). Lower limit of detection (LLOD) was determined as the mean of repeated measures of blank + 2.5 SD, either based on ten repeated blanks or the most representative calibration curve of the six available curves (each curve having two blank measurements). Intraassay variation was determined by 16 repeating wells of high control. Ten samples were run double. Fifteen samples from three individuals were used for validating the concentration of neuroinflammatory markers over time and quantified with ten minutes intervals.

*Estimation with the censored log-normal model*

Censored observations are typical in time-to-event analyses such as survival analyses so software for parametric survival models supporting Gaussian or log-normal distributions to be applied to estimate the censored log-normal model (5,6). We use the survival package in R (7). While survival analyses are typically right censored (death is only known to occur *after* a censoring date) censored concentration measurements are left censored since the concentration is only known to *smaller* than some LLOD/Q. However, the same methodology applies and can be used for unbiased estimation of effects almost irrespective of the degree of censoring.

Let $y_{i}$ be the $i$’th cytokine measurement, then we consider a Gaussian linear model for the log cytokine measurement of the form

$$\mathsf{E}\left[ \log Y_{i}|x_{i} \right]=\mu_{i}=\alpha+\mathrm{group}_{i}+\mathrm{sex}_{i}+\mathrm{age}_{i}+\mathrm{plate}_{i}, \log Y_{i}\sim N\left( \mu_{i},\sigma^{2} \right), i=1,\ldots,n$$

where $x_{i}$ represent the variables on group, sex, age and plate and $\alpha$ is an intercept.

However, $y_{i}$ is only fully observed if $\log y_{i}$ is greater than the point of censoring, $\tau$ (e.g., LLOD or LLOQ). The log-likelihood function therefore takes the following form (8):

$$\mathcal{l}\left( \mu,\sigma^{2};y,x \right)=\sum_{i=1}^{n} I\left( \log y_{i}\leq\tau\right)\log\Phi\left( \tau;\mu_{i},\sigma^{2} \right)+I\left( \log y_{i}>\tau\right)\log\phi\left( \log y_{i};\mu_{i},\sigma^{2} \right)$$

where $\Phi\left( \cdot\right)$ is the standard normal distribution function and $\phi\left( \cdot\right)$ is the standard normal probability mass function. The model parameters are then estimated by maximizing the log-likelihood function.

The censored log-normal model can be estimated in R for each cytokine using the survival package with the following code.

library(survival)
fm <- survreg(Surv(time=Y, event=(Y > tau), type="left") ~
 group + sex + age + plate, dist='lognormal', data=cytokine_data)

The code assumes the data frame cytokine_data contains the variables Y (cytokine measurement), group (factor variable), sex (factor variable), age (numeric/continuous variable), plate (factor variable) and that the point of censoring tau has been defined.

**References**

1. Turner MD, Nedjai B, Hurst T, Pennington DJ. Cytokines and chemokines: At the crossroads of cell signalling and inflammatory disease. Vol. 1843, Biochimica et biophysica acta. Netherlands; 2014. p. 2563–82.

2. Lacy P, Stow JL. Cytokine release from innate immune cells: association with diverse membrane trafficking pathways. Blood. 2011 Jul;118(1):9–18.

3. Feghali CA, Wright TM. Cytokines in acute and chronic inflammation. Front Biosci. 1997 Jan;2:d12-26.

4. Himmerich H, Patsalos O, Lichtblau N, Ibrahim MAA, Dalton B. Cytokine Research in Depression: Principles, Challenges, and Open Questions. Front psychiatry. 2019;10:30.

5. Lynn HS. Maximum likelihood inference for left-censored HIV RNA data. Stat Med. 2001 Jan;20(1):33–45.

6. Uh H-W, Hartgers FC, Yazdanbakhsh M, Houwing-Duistermaat JJ. Evaluation of regression methods when immunological measurements are constrained by detection limits. BMC Immunol. 2008 Oct;9:59.

7. Therneau T. A Package for Survival Analysis in R. R Packag version 32-13 [Internet]. 2021; Available from: https://cran.r-project.org/package=survival

8. Pawitan Y. In All Likelihood Statistical Modelling and Inference Using Likelihood. 2001.
